# Early-life and health behaviour influences on lung function in early adulthood

**DOI:** 10.1183/13993003.01316-2020

**Published:** 2023-03-02

**Authors:** Osama Mahmoud, Raquel Granell, Gabriela P. Peralta, Judith Garcia-Aymerich, Deborah Jarvis, John Henderson, Jonathan Sterne

**Affiliations:** 1 Dept of Mathematical Sciences, University of Essex, Colchester, UK; 2 Population Health Sciences, Bristol Medical School, University of Bristol, Bristol, UK; 3 Dept of Applied Statistics, Helwan University, Cairo, Egypt; 4 MRC Integrative Epidemiology Unit (IEU), Bristol Medical School, University of Bristol, Bristol, UK; 5 Epidemiology, Biostatistics and Prevention Institute (EBPI), University of Zurich, Zurich, Switzerland; 6 ISGlobal, Barcelona, Spain; 7 Universitat Pompeu Fabra (UPF), Barcelona, Spain; 8 CIBER Epidemiología y Salud Pública (CIBERESP), Barcelona, Spain; 9 National Heart and Lung Institute, Imperial College, London, UK; 10 MRC-PHE Centre for Environment and Health, Imperial College, London, UK; † Deceased

## Abstract

**Rationale:**

Early-life exposures may influence lung function at different stages of the life course. However, the relative importance of characteristics at different stages of infancy and childhood are unclear.

**Objectives:**

To examine the associations and relative importance of early-life events on lung function at age 24 years.

**Methods:**

We followed 7545 children from the Avon Longitudinal Study of Parents and Children from birth to 24 years. Using previous knowledge, we classified an extensive list of putative risk factors for low lung function, covering sociodemographic, environmental, lifestyle and physiological characteristics, according to timing of exposure: 1) demographic, maternal and child; 2) perinatal; 3) postnatal; 4) early childhood; and 5) adolescence characteristics. Lung function measurements (forced vital capacity (FVC), forced expiratory volume in 1 s (FEV_1_), FEV_1_/FVC and forced expiratory flow at 25–75% of FVC) were standardised for sex, age and height. The proportion of the remaining variance explained by each characteristic was calculated. The association and relative importance (RI) of each characteristic for each lung function measure was estimated using linear regression, adjusted for other characteristics in the same and previous categories.

**Results:**

Lower maternal perinatal body mass index (BMI), lower birthweight, lower lean mass and higher fat mass in childhood had the largest RI (0.5–7.7%) for decreased FVC. Having no siblings, lower birthweight, lower lean mass and higher fat mass were associated with decreased FEV_1_ (RI 0.5–4.6%). Higher lean mass and childhood asthma were associated with decreased FEV_1_/FVC (RI 0.6–0.8%).

**Conclusions:**

Maternal perinatal BMI, birthweight, childhood lean and fat mass and early-onset asthma are the factors in infancy and childhood that have the greatest influence on early-adult lung function.

## Abstract 2

**Perinatal characteristics (*e.g.* birth weight) and childhood characteristics (*e.g.* lean mass, fat mass and asthma at primary school age) had the most influence on lung function in early adulthood** https://bit.ly/3TggF1Q

## Introduction

Lung development commences in early gestation and lung growth continues up until early adulthood (20–25 years of age) when a physiological plateau in lung function is attained [1–3]. Low maximally attained lung function is associated with higher risk and earlier onset of chronic obstructive pulmonary disease, higher susceptibility to cardiorespiratory morbidity and all-cause mortality in adulthood [4]. Based on many experimental and epidemiological observations of immunological and pulmonary development, characteristics of early life, including the prenatal period, appear likely to have a major influence on lung function in adult life [5–7]. Understanding the role of early development and exposure to environmental and health behaviour characteristics in attained lung function in early adulthood may provide insights into later development of lung function impairment, explain growth-related differences in their risks and identify targets for early intervention [8–12].

Numerous studies have investigated variables that might influence lung function growth and related respiratory diseases in childhood and adolescence. Identified variables include prenatal stress [6]; mode of delivery [13]; maternal diet [14]; history of child early feeding [14, 15]; infancy peak weight velocity [16]; exposure to pollutions [17–19] and allergens [20, 21] in early childhood; and the roles of respiratory viral infections [22], physical activity [23], body composition [24] and pubertal growth [25–27]. Most studies have focused on one or a few characteristics, but variations in lung function are likely due to simultaneous effects of several characteristics [2, 28]. Few studies have investigated the simultaneous association of several characteristics with lung function in childhood and adolescence [29–31]. But none to our knowledge has either combined sociodemographic, environmental, lifestyle and physiological characteristic risk factors measured at different stages of early life-course or investigated their simultaneous associations with lung function in early adulthood, around the attainment of the physiological plateau in lung function.

**FIGURE 1:**
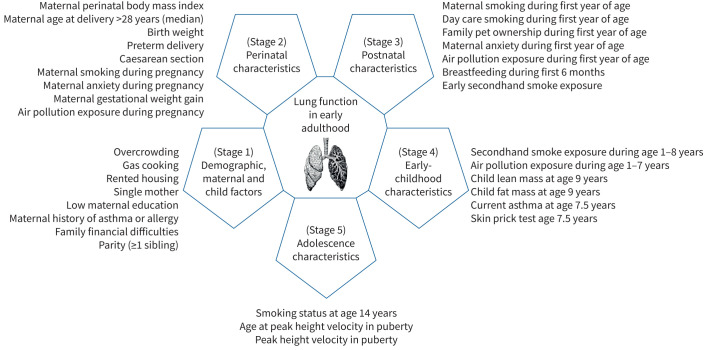
Characteristics examined for association and relative importance with lung function at age 24 years (detailed description presented in table 1).

We analysed data from a large population-based British birth cohort to investigate associations of a wide range of characteristics covering early-life events through adolescence with lung function in early-adult life, around the time of expected peak lung function attainment. Our aims were to examine numerous characteristics (figure 1) to identify those independently associated with lung function in early adulthood, to assess proportions of explained variations in lung function parameters attributed to each characteristic, and hence to derive the relative importance (RI) of the characteristics for early-adult lung function.

## Methods

### Study design, setting and population

**FIGURE 2:**
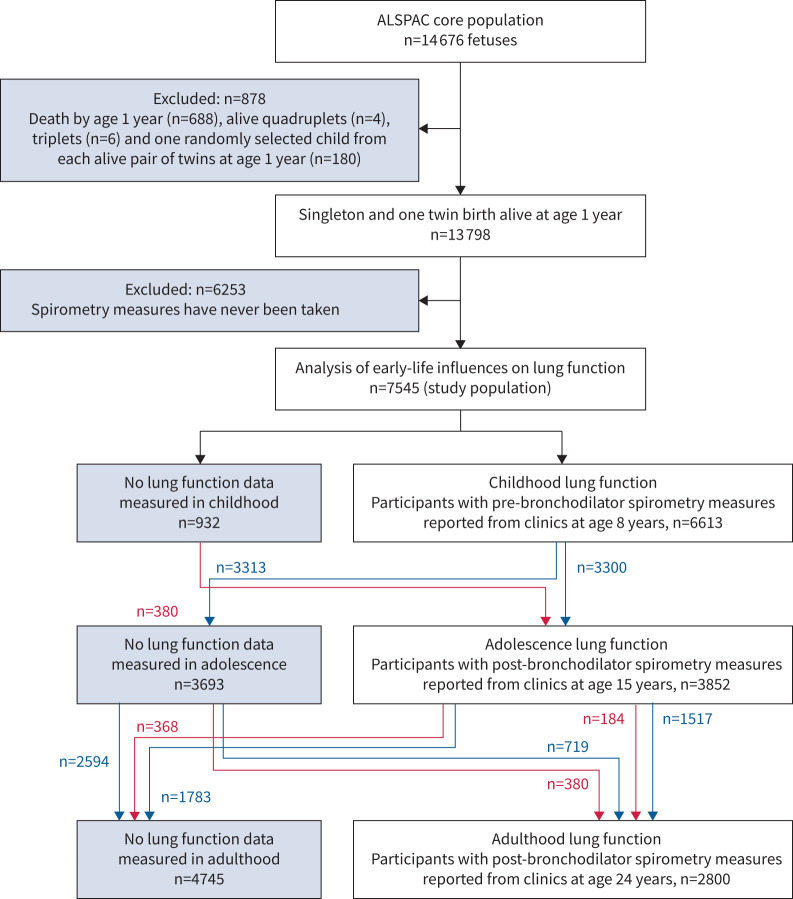
Flow chart of study participants. The blue and red arrows refer to different follow-up paths for spirometry clinics at ages 8, 15 and 24 years. For example, the blue arrow from ‘Childhood lung function’ to ‘Adolescence lung function’ boxes represents participants (n=3300) whose lung function was measured at both clinics; of those, n=1517 participants had lung function measurements in adulthood but n=1783 had not (the two blue arrows coming out from the box ‘Adolescence lung function’). ALSPAC: Avon Longitudinal Study of Parents and Children.

We studied participants in the Avon Longitudinal Study of Parents and Children (ALSPAC), a British population-based birth cohort. The study protocol has been presented previously [32–34], and a detailed description is provided in the supplementary material. Briefly, 14 541 pregnant women resident in Avon, UK, with expected delivery dates between 1 April 1991 and 31 December 1992 were recruited, and their 14 062 live-born children were monitored prospectively. The 7545 participants who had lung function measured at least once at ages 8, 15 and 24 years were included in this study. A flow chart of the study participants is provided in figure 2. Additional details are in the supplementary material.

### Lung function

Spirometry was performed according to American Thoracic Society/European Respiratory Society criteria [35, 36] by trained fieldworkers in a research clinic at ages 8, 15 and 24 years. All flow-volume curves were inspected *post hoc* for quality assurance by JH. Lung function at ages 15 and 24 years was measured before and 15 min after receiving 400 µg of salbutamol [37, 38]. The highest measurement of each lung function parameter, *i.e.* forced vital capacity (FVC), forced expiratory volume in 1 s (FEV_1_) and forced expiratory flow at 25–75% of FVC (FEF_25–75%_), among the best three technically acceptable flow-volume curves was used for analyses. Standardised post-bronchodilator lung function scores (sd scores adjusted for sex, age and height) at age 24 years were used as the outcomes. Our sd scores were not adjusted for race because the majority (96.3%) of participants in our study population (n=7545) were from the same ethnic group (described as white).

### Description of characteristics

We considered sociodemographic, environmental, lifestyle and physiological characteristics based on a review of the literature [1, 28, 39], including previous ALSPAC publications [10, 11, 23–25, 40]. Figure 1 shows an overview of the investigated characteristics, and table 1 provides details of their descriptions. There were 33 characteristics identified and clustered into five life-course stages: 1) demographic, maternal and child; 2) perinatal; 3) postnatal; 4) early childhood; and 5) adolescence. Additional details are in the supplementary material.

**TABLE 1:** Description of investigated factors grouped in five life-course stages

**Stage**	**Factor**	**Description**	**Assessment**
**Demographic, maternal and child characteristics**	Overcrowding	Positive if home has >0.75 persons per room	Questionnaires sent to mother during pregnancy or 3–12 months after delivery
	Gas cooking	Yes or no (baseline)	As above
	Rented housing	Yes or no (baseline)	As above
	Single mother	Yes or no (baseline)	As above
	Low maternal education	Positive if mother educated to school leaving certificate at 16 years (GCE level in UK) or lower	As above
	Maternal history of asthma or allergy	Yes or no (baseline)	As above
	Family financial difficulties	Positive if financial difficulties reported at all three assessment points	Asked at 32 weeks in pregnancy, and 8 and 21 months after delivery (questionnaire-based)
	Parity	Positive if the child has ≥1 sibling	At birth (questionnaire)
**Perinatal characteristics**	Maternal perinatal (early pregnancy) BMI	Continuous (kg·m^−2^)	Measured at 12 weeks’ gestation
	Maternal age at delivery	Dichotomised as ≤28 and >28 years (median age served as the cut-off)	Using delivery healthcare records
	Birthweight	Continuous (kg)	As above
	Preterm delivery	Positive if gestation <37 weeks	As above
	Caesarean section	Yes or no (baseline)	As above
	Maternal smoking during pregnancy	Yes or no (baseline)	Questionnaires sent at 32 weeks’ gestation
	Maternal anxiety during pregnancy^#^	Yes or no (baseline)	As above
	Maternal gestational weight gain	Continuous (kg·week^−1^)	Mean weight gain at 0–18 and 18–28 weeks in pregnancy
	Air pollution exposure during pregnancy	Continuous (µg·m^−3^)	Average of daily concentration of source-specific PM_10_
**Postnatal characteristics**	Maternal smoking during first year of age	Yes or no (baseline)	Questionnaires sent from 3 to 15 months after birth
	Day care attendance during first year of age	Yes or no (baseline)	As above
	Family pet ownership during first year of age	Yes or no (baseline)	As above
	Maternal anxiety during first year of age^#^	Yes or no (baseline)	As above
	Air pollution exposure during first year of age	Continuous (µg·m^−3^)	Average of daily concentration of source-specific PM_10_ measured at age 6 and 12 months
	Breastfeeding during first 6 months	Yes or no (baseline)	Questionnaire-based, sent from 3 to 15 months after birth
	Early secondhand smoke exposure	Yes or no (baseline)	As above
**Early-childhood characteristics**	Secondhand smoke exposure during age 1–8 years	Positive if exposure to secondhand smoke at home reported at least in one questionnaire during age 1–8 years	Annual questionnaires sent from age 1–6 and at 8 years
	Air pollution exposure during age 1–7 years	Continuous (µg·m^−3^)	Cumulative concentration of source-specific PM_10_ assessed annually during age 1–7 years
	Child lean mass at age 9 years	Continuous (kg), residual after adjustment for gender and height	Measured at focus clinic and expressed as residuals from a linear regression of each on gender, height and height squared
	Child fat mass at age 9 years	Continuous (kg/2), residual after adjustment for gender and height	As above, residual fat mass was divided by 2 (supplementary methods)
	Current asthma at age 7.5 years	Yes or no (baseline)	Questionnaire-based at age 7.5 years
	Allergic sensitisation (skin prick test) at age 7.5 years	Positive if any of skin prick tests for grass, cat or house dust mite reported positive result	Measured using cut-off weal for positivity ≥2 mm
**Adolescence characteristics**	Smoking status at age 14 years	Positive if smoked at least one cigarette	Questionnaire-based at age 14 years
	Age at peak height velocity in puberty^¶^	Continuous (years)	Derived using mixed-effects models for repeated height measurements from age 5 to 20 years [25]
	Peak height velocity in puberty	Continuous (cm·year^−1^)	As above

BMI: body mass index; PM_10_: particulate matter ≤10 µm in diameter. ^#^: anxiety was measured using the validated self-report Crown–Crisp Experiential Index which ranges from 0 (not anxious) to 16 (very anxious) [41]; maternal anxiety scores were not normally distributed and therefore were converted into 1st quartile (0–2), 2nd quartile (3–4), 3rd quartile (5–7) and 4th quartile (8–16), with anxious mothers defined as being in the 4th quartile; ^¶^: peak height velocity is defined as the maximum of the first derivative of individual height growth trajectories, fitted using nonlinear mixed-effects models, from age 5–20 years [25].

### Statistical analysis

We compared the characteristics of the study population (n=7545) with those of the original cohort (singleton and one of each twin birth alive at age 1 year, n=13 798). Participants in the study population with (n=2800) and without (n=4745) lung function measurements at age 24 years were also compared.

To increase power and minimise selection bias, multiple imputation (20 imputed datasets) by chained equations was performed to impute missing data of investigated characteristics and lung function outcomes at age 24 years [42]. Imputation models included all predictor variables analysed as well as measures of lung function at ages 8 and 15 years. We compared the characteristics of the study population using observed and imputed datasets to assess the empirical distributions of the examined characteristics and the lung function outcomes before and after the imputation. To assess the robustness of our findings, we repeated our analyses using data from only the participants with measured (non-imputed) lung function at age 24 years.

We estimated associations with lung function at age 24 years according to temporal ordering of life-course stages, starting with demographic, maternal and child characteristics. First, mutually adjusted associations of these characteristics with each lung function parameter at age 24 years were estimated using multivariable linear regression models fitted to each of the 20 imputed datasets, with results combined using Rubin's rules [43]. We then estimated mutually adjusted associations of perinatal characteristics (our second stage), additionally adjusting for potential confounding by the characteristics from the previous stage for which the p-value was <0.1. This process continued by estimating associations of characteristics for the next three stages, adjusting for potential confounding by characteristics with p≤0.1 from previous stages.

#### Relative importance derivation

For each stage, we calculated the increment in the explained variance (R^2^) in lung function at age 24 years when all characteristics in the stage were added to a model including the retained characteristics (those with p≤0.1) from previous stages, if any. This has been referred to as stage incremental R^2^. Within each stage, we derived the increment in R^2^ attributed to each characteristic (characteristic incremental R^2^) by adding the characteristics one by one to a model. A characteristic's contribution to a stage incremental R^2^ depends on the order in which the characteristic is added to the model among other characteristics in the same stage. A characteristic appears to contribute more to a stage's incremental R^2^ when it is added first due to correlations between characteristics in the same stage. Therefore, we derived incremental R^2^ for each characteristic by averaging its contribution to the stage incremental R^2^ over all its possible orderings among the set of characteristics in its stage.

The RI of a characteristic is defined as its incremental R^2^ when all characteristics in the same stage as the considered characteristic were added to the model including the retained characteristics from previous stages. It is then an estimate of the proportion of variance in lung function at age 24 years explained by the characteristic using our model setup. This derivation of RI implies that the sum of the RI values of all characteristics within a stage equals the incremental R^2^ of this stage.

All analyses were conducted using the statistical software R, version 3.5.0 (www.r–project.org). RI was derived using the Lindeman, Merenda and Gold method [44] from the relaimpo R package [45]. Further details on the methods are provided in the supplementary material.

## Results

Among 7545 participants with at least one spirometry measurement at ages 8, 15 or 24 years, 51% were female, 18.3% had a single mother, 57.5% had a mother with a low educational level, 47.3% had a maternal history of asthma or allergy, 3.4% had family financial difficulties, 53.7% had siblings, 5.4% were born preterm, 10.5% were born with caesarean section, and 20.9% and 30.2% had maternal smoking and anxiety during pregnancy, respectively (supplementary table S1). Spirometry measurements were taken for 88%, 51% and 37% of participants at ages 8, 15 and 24 years, respectively (figure 2). The summary statistics for investigated characteristics showed similar results for the original ALSPAC cohort and our study population (supplementary table S2), for observed and imputed data (supplementary table S1) and for participants with and without lung function measurements at age 24 years (supplementary table S3). The summary statistics for lung function outcomes at age 24 years were similar in observed and imputed data (supplementary table S4). The amount of missing data for each characteristic and lung function measurement in the study population is depicted in supplementary figure S1.

sd scores of lung function measurements at age 24 years standardised for sex, age and height showed positive linear correlations with sd scores of lung function measured earlier at ages 8 years (coefficients ranged between 0.50 and 0.51 across different lung function parameters) and 15 years (range 0.46–0.48) (supplementary table S5). This degree of correlation enabled missing lung function data at age 24 years to be imputed by including earlier measurements of lung function in the imputation models.

### Associations with lung function in early adulthood

**FIGURE 3:**
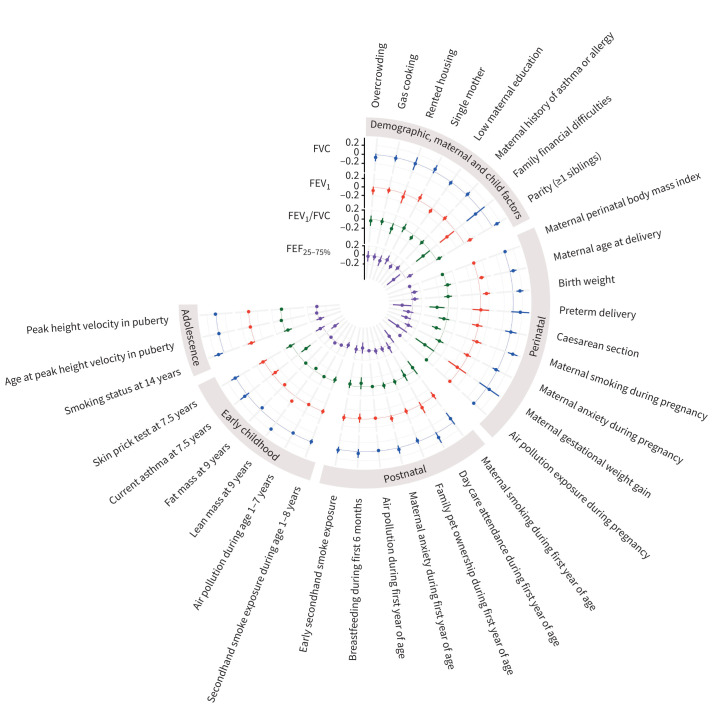
Circular plot of characteristics’ association (point estimates and 95% confidence intervals) with lung function parameters at age 24 years for our study population (n=7545). The raw data used for generating this plot are reported in tables 2–5. FVC: forced vital capacity; FEV_1_: forced expiratory volume in 1 s; FEF_25–75%_: forced expiratory flow at 25–75% of FVC.

Among demographic, maternal and child characteristics, parity was positively associated with higher FVC (0.12 sd, 95% CI 0.05–0.20) and FEV_1_ (0.16 sd, 95% CI 0.09–0.23), and family financial difficulties with low FEV_1_ (−0.25 sd, 95% CI −0.46– −0.03). Associations of parity with FVC and FEV_1_ were slightly attenuated (0.10 sd, 95% CI 0.03–0.17, and 0.14 sd, 95% CI 0.06–0.21, respectively) when additionally adjusted for birthweight. Among perinatal characteristics, higher birthweight was associated with higher FVC (0.16 sd, 95% CI 0.08–0.23) and FEV_1_ (0.15 sd, 95% CI 0.07–0.23) per kg, and higher perinatal body mass index (BMI) and maternal smoking during pregnancy were associated with higher FVC (0.02 sd, 95% CI 0.01–0.03 per kg·m^−2^, and 0.18 sd, 95% CI 0.07–0.29, respectively). Higher maternal age at delivery was associated with higher FEV_1_ (0.09 sd, 95% CI 0.03–0.15). Among early-childhood characteristics, higher lean mass (LM) and lower fat mass (FM) at age 9 years were associated with higher FVC (0.18 sd, 95% CI 0.16–0.20 per kg, and −0.05 sd, 95% CI −0.07– −0.03 per kg/2, respectively) and FEV_1_ (0.14 sd, 95% CI 0.12–0.16 per kg, and −0.05 sd, 95% CI −0.06– −0.03 per kg/2, respectively). Among adolescence characteristics, smoking at age 14 years was associated with higher FVC (0.13 sd, 95% CI 0.03–0.23), with no evidence of an association with FEV_1_ (0.09 sd, 95% CI −0.01–0.18) (figure 3, tables 2 and 3).

**TABLE 2:** Adjusted association and RI of early-life characteristics with sd scores of FVC (scores adjusted for sex, age and height) at age 24 years (n=7545)

**Stage**	**Factor**	**Adjusted difference in sd scores of FVC (95% CI)** ^#^	**p-value**	**Incremental R^2^ (%)** ^¶^	**RI (%)**	**Retained R^2^** ^+^
**Demographic, maternal and child characteristics**	Overcrowding	−0.060 (−0.145–0.024)	0.164	0.61	0.047	0.32
	Gas cooking	−0.015 (−0.086–0.056)	0.683		0.020	
	Rented housing	−0.010 (−0.159–0.138)	0.892		0.040	
	Single mother	0.056 (−0.044–0.155)	0.277		0.033	
	Low maternal education	−0.020 (−0.081–0.041)	0.529		0.020	
	Maternal history of asthma or allergy	0.026 (−0.043–0.095)	0.460		0.033	
	Family financial difficulties	−0.141 (−0.395–0.112)	0.282		0.095	
	Parity (≥1 siblings)	0.123 (0.050–0.196)	0.002		0.318	
**Perinatal characteristics**	Maternal perinatal body mass index (kg·m^−2^)	0.020 (0.009–0.032)	0.001	1.98	0.631	2.04
	Maternal age at delivery >28 years (the median)	0.075 (0.003–0.147)	0.047		0.113	
	Birthweight (kg)	0.157 (0.079–0.234)	2×10^−4^		0.536	
	Preterm delivery	0.161 (−0.036–0.357)	0.117		0.076	
	Caesarean section	0.010 (−0.117–0.137)	0.878		0.029	
	Maternal smoking during pregnancy	0.178 (0.068–0.288)	0.003		0.440	
	Maternal anxiety during pregnancy	−0.029 (−0.112–0.055)	0.505		0.032	
	Maternal gestational weight gain (kg·week^−1^)	0.066 (−0.196–0.327)	0.624		0.036	
	Air pollution exposure during pregnancy (μg·m^−3^)	−0.009 (−0.020–0.003)	0.139		0.090	
**Perinatal characteristics**	Maternal smoking during first year of age	−0.100 (−0.258–0.059)	0.226	0.38	0.087	2.04
	Day care attendance during first year of age	0.107 (−0.026–0.241)	0.120		0.085	
	Family pet ownership during first year of age	−0.026 (−0.097–0.044)	0.467		0.026	
	Maternal anxiety during first year of age	0.040 (−0.050–0.131)	0.384		0.047	
	Air pollution during first year of age (μg·m^−3^)	−0.010 (−0.023–0.002)	0.118		0.077	
	Breastfeeding during first 6 months	0.042 (−0.054–0.138)	0.397		0.046	
	Early secondhand smoke exposure	0.016 (−0.058–0.089)	0.677		0.015	
**Early-childhood characteristics**	Secondhand smoke exposure during age 1–8 years	−0.003 (−0.062–0.056)	0.921	8.58	0.008	10.48
	Air pollution during 1–7 years of age (μg·m^−3^)	−0.010 (−0.019– −0.001)	0.034		0.172	
	Lean mass at age 9 years (sd score)	0.180 (0.159–0.201)	1×10^−16^		7.707	
	Fat mass at age 9 years (sd score)	−0.051 (−0.073– −0.028)	1×10^−4^		0.556	
	Current asthma at 7.5 years	0.064 (−0.056–0.184)	0.304		0.104	
	Skin prick test at 7.5 years	0.015 (−0.083–0.113)	0.766		0.030	
**Adolescence characteristics**	Smoking status at 14 years	0.130 (0.031–0.228)	0.014	0.38	0.337	10.82
	Age at peak height velocity in puberty (years)	−0.003 (−0.028–0.022)	0.822		0.011	
	Peak height velocity in puberty (cm·year^−1^)	0.012 (−0.012–0.036)	0.319		0.033	

RI: relative importance (proportion of explained variation in lung function attributed to each variable, averaging over all its possible orderings among characteristics in same stage); FVC: forced vital capacity. ^#^: adjusted for all variables in same stage in addition to characteristics from previous stages that yield p-value ≤0.10; ^¶^: for variables in the corresponding stage; ^+^: total R^2^ for retained variables (with p≤0.10) from previous stages and corresponding stage.

**TABLE 3:** Adjusted association and RI of early-life characteristics with sd scores of FEV_1_ (scores adjusted for sex, age and height) at age 24 years (n=7545)

**Stage**	**Factor**	**Adjusted difference in sd scores of FEV** **_1_ (95% CI)** ^#^	**p-value**	**Incremental R^2^ (%)** ^¶^	**RI (%)**	**Retained R^2^** ^+^
**Demographic, maternal and child characteristics**	Overcrowding	−0.079 (−0.173–0.015)	0.107	1.21	0.104	0.89
	Gas cooking	−0.021 (−0.091–0.048)	0.552		0.022	
	Rented housing	−0.075 (−0.236–0.085)	0.366		0.134	
	Single mother	0.067 (−0.036–0.169)	0.208		0.039	
	Low maternal education	−0.067 (−0.133– −0.001)	0.050		0.141	
	Maternal history of asthma or allergy	0.017 (−0.054–0.088)	0.641		0.027	
	Family financial difficulties	−0.246 (−0.459– −0.033)	0.029		0.220	
	Parity (≥1 siblings)	0.161 (0.089–0.233)	5×10^−5^		0.524	
**Perinatal characteristics**	Maternal perinatal body mass index (kg·m^−2^)	0.006 (−0.004–0.015)	0.263	1.06	0.086	1.59
	Maternal age at delivery >28 years (the median)	0.086 (0.026–0.147)	0.006		0.170	
	Birthweight (kg)	0.147 (0.066–0.229)	0.001		0.529	
	Preterm delivery	0.011 (−0.184–0.206)	0.909		0.075	
	Caesarean section	−0.025 (−0.148–0.097)	0.689		0.027	
	Maternal smoking during pregnancy	0.066 (−0.048–0.180)	0.266		0.074	
	Maternal anxiety during pregnancy	−0.021 (−0.112–0.071)	0.657		0.038	
	Maternal gestational weight gain (kg·week^−1^)	−0.076 (−0.329–0.177)	0.558		0.025	
	Air pollution exposure during pregnancy (μg·m^−3^)	−0.005 (−0.015–0.006)	0.372		0.035	
**Perinatal characteristics**	Maternal smoking during first year of age	−0.013 (−0.114–0.087)	0.793	0.27	0.025	1.59
	Day care attendance during first year of age	0.080 (−0.067–0.228)	0.289		0.057	
	Family pet ownership during first year of age	−0.002 (−0.075–0.071)	0.966		0.013	
	Maternal anxiety during first year of age	0.062 (−0.018–0.141)	0.134		0.076	
	Air pollution during first year of age (μg·m^−3^)	−0.008 (−0.020–0.005)	0.231		0.048	
	Breastfeeding during first 6 months	−0.018 (−0.125–0.088)	0.737		0.032	
	Early secondhand smoke exposure	0.009 (−0.068–0.085)	0.826		0.019	
**Early-childhood characteristics**	Secondhand smoke exposure during age 1–8 years	0.002 (−0.057–0.061)	0.948	5.26	0.011	6.63
	Air pollution during 1–7 years of age (μg·m^−3^)	−0.007 (−0.016–0.003)	0.167		0.096	
	Lean mass at age 9 years (sd score)	0.140 (0.117–0.163)	3×10^−15^		4.579	
	Fat mass at age 9 years (sd score)	−0.045 (−0.063– −0.026)	3×10^−5^		0.465	
	Current asthma at 7.5 years	−0.072 (−0.171–0.026)	0.158		0.072	
	Skin prick test at 7.5 years	0.027 (−0.076–0.130)	0.612		0.032	
**Adolescence characteristics**	Smoking status at 14 years	0.088 (−0.002–0.178)	0.063	0.19	0.162	6.79
	Age at peak height velocity in puberty (years)	0.009 (−0.019–0.037)	0.542		0.019	
	Peak height velocity in puberty (cm·year^−1^)	−0.001 (−0.026–0.024)	0.921		0.012	

RI: relative importance (proportion of explained variation in lung function attributed to each variable, averaging over all its possible orderings among characteristics in same stage); FEV_1_: forced expiratory volume in 1 s. ^#^: adjusted for all variables in same stage in addition to characteristics from previous stages that yield p-value ≤0.10; ^¶^: for variables in the corresponding stage; ^+^: total R^2^ for retained variables (with p≤0.10) from previous stages and corresponding stage.

Among demographic, maternal and child characteristics, lower maternal education was associated with lower FEV_1_/FVC (−0.08 sd, 95% CI −0.14– −0.02) and FEF_25–75%_ (−0.07 sd, 95% CI −0.13– −0.01), family financial difficulties with lower FEF_25–75%_ (−0.24 sd, 95% CI −0.42– −0.06) and parity with higher FEF_25–75%_ (0.11 sd, 95% CI 0.03–0.18). Among perinatal characteristics, preterm delivery was associated with lower FEV_1_/FVC (−0.25 sd, 95% CI −0.41– −0.08) and FEF_25–75%_ (−0.23 sd, 95% CI −0.43– −0.02), and higher maternal perinatal BMI and maternal smoking during pregnancy with lower FEV_1_/FVC (−0.02 sd, 95% CI −0.03– −0.01, and −0.17 sd, 95% CI −0.27– −0.07, respectively). Among early-childhood characteristics, higher LM was associated with lower FEV_1_/FVC (−0.05 sd, 95% CI −0.08– −0.03) but higher FEF_25–75%_ (0.04 sd, 95% CI 0.01–0.06), and asthma at age 7.5 years with lower FEV_1_/FVC (−0.22 sd, 95% CI −0.34– −0.09) and FEF_25–75%_ (−0.24 sd, 95% CI −0.34– −0.14) (figure 3, tables 4 and 5).

**TABLE 4:** Adjusted association and RI of early-life characteristics with sd scores of FEV_1_/FVC (scores adjusted for sex, age and height) at age 24 years (n=7545)

**Stage**	**Factor**	**Adjusted difference in sd scores of FEV_1_/FVC** **(95% CI)**^#^	**p-value**	**Incremental R^2^ (%)** ^¶^	**RI (%)**	**Retained R^2^** ^+^
**Demographic, maternal and child characteristics**	Overcrowding	−0.033 (−0.146–0.081)	0.574	0.79	0.067	0.45
	Gas cooking	−0.013 (−0.091–0.064)	0.736		0.025	
	Rented housing	−0.107 (−0.233–0.020)	0.106		0.194	
	Single mother	0.019 (−0.084–0.123)	0.715		0.036	
	Low maternal education	−0.079 (−0.142– −0.015)	0.017		0.184	
	Maternal history of asthma or allergy	−0.011 (−0.079–0.057)	0.751		0.018	
	Family financial difficulties	−0.194 (−0.386– −0.002)	0.052		0.155	
	Parity (≥1 siblings)	0.069 (−0.003–0.142)	0.067		0.106	
**Perinatal characteristics**	Maternal perinatal body mass index (kg·m^−2^)	−0.021 (−0.032– −0.010)	0.001	1.66	0.543	1.85
	Maternal age at delivery >28 years (the median)	0.027 (−0.047–0.101)	0.480		0.049	
	Birthweight (kg)	−0.001 (−0.086–0.084)	0.980		0.046	
	Preterm delivery	−0.247 (−0.413– −0.082)	0.005		0.291	
	Caesarean section	−0.064 (−0.183–0.055)	0.298		0.089	
	Maternal smoking during pregnancy	−0.173 (−0.273– −0.072)	0.002		0.495	
	Maternal anxiety during pregnancy	0.003 (−0.095–0.100)	0.953		0.039	
	Maternal gestational weight gain (kg·week^−1^)	−0.229 (−0.476–0.019)	0.075		0.075	
	Air pollution exposure during pregnancy (μg·m^−3^)	0.004 (−0.007–0.016)	0.441		0.035	
**Perinatal characteristics**	Maternal smoking during first year of age	−0.073 (−0.251–0.105)	0.428	0.31	0.067	1.85
	Day care attendance during first year of age	−0.030 (−0.171–0.110)	0.672		0.022	
	Family pet ownership during first year of age	0.012 (−0.054–0.079)	0.714		0.012	
	Maternal anxiety during first year of age	0.021 (−0.049–0.091)	0.554		0.015	
	Air pollution during first year of age (μg·m^−3^)	0.001 (−0.014–0.016)	0.923		0.023	
	Breastfeeding during first 6 months	−0.077 (−0.208–0.054)	0.259		0.123	
	Early secondhand smoke exposure	−0.036 (−0.126–0.054)	0.433		0.047	
**Early-childhood characteristics**	Secondhand smoke exposure during age 1–8 years	−0.036 (−0.109–0.037)	0.337	1.67	0.046	3.33
	Air pollution during 1–7 years of age (μg·m^−3^)	0.003 (−0.007–0.012)	0.559		0.031	
	Lean mass at age 9 years (sd score)	−0.054 (−0.076– −0.033)	6×10^−6^		0.843	
	Fat mass at age 9 years (sd score)	0.001 (−0.019–0.021)	0.925		0.071	
	Current asthma at 7.5 years	−0.217 (−0.342– −0.092)	0.002		0.636	
	Skin prick test at 7.5 years	0.035 (−0.069–0.139)	0.510		0.038	
**Adolescence characteristics**	Smoking status at 14 years	−0.062 (−0.152–0.028)	0.182	0.21	0.100	3.44
	Age at peak height velocity in puberty (years)	0.021 (−0.003–0.045)	0.096		0.064	
	Peak height velocity in puberty (cm·year^−1^)	−0.018 (−0.039–0.003)	0.099		0.048	

RI: relative importance (proportion of explained variation in lung function attributed to each variable, averaging over all its possible orderings among characteristics in same stage); FEV_1_: forced expiratory volume in 1 s; FVC: forced vital capacity. ^#^: adjusted for all variables in same stage in addition to characteristics from previous stages that yield p-value ≤0.10; ^¶^: for variables in the corresponding stage; ^+^: total R^2^ for retained variables (with p≤0.10) from previous stages and corresponding stage.

**TABLE 5:** Adjusted association and RI of early-life characteristics with sd scores of FEF_25–75%_ (scores adjusted for sex, age and height) at age 24 years (n=7545)

**Stage**	**Factor**	**Adjusted difference in sd scores of FEF_25–75%_ (95% CI)** ^#^	**p-value**	**Incremental R^2^ (%)** ^¶^	**RI (%)**	**Retained R^2^** ^+^
**Demographic, maternal and child characteristics**	Overcrowding	−0.029 (−0.143–0.085)	0.625	0.84	0.050	0.61
	Gas cooking	−0.021 (−0.098–0.056)	0.599		0.029	
	Rented housing	−0.069 (−0.190–0.052)	0.271		0.102	
	Single mother	0.014 (−0.093–0.120)	0.802		0.034	
	Low maternal education	−0.073 (−0.134– −0.012)	0.020		0.152	
	Maternal history of asthma or allergy	0.003 (−0.065–0.071)	0.939		0.016	
	Family financial difficulties	−0.238 (−0.420– −0.057)	0.012		0.204	
	Parity (≥1 siblings)	0.108 (0.034–0.181)	0.006		0.254	
**Perinatal characteristics**	Maternal perinatal body mass index (kg·m^−2^)	−0.001 (−0.011–0.009)	0.859	1.03	0.019	1.07
	Maternal age at delivery >28 years (the median)	0.066 (−0.003–0.135)	0.064		0.130	
	Birthweight (kg)	0.053 (−0.025–0.131)	0.187		0.175	
	Preterm delivery	−0.227 (−0.429– −0.024)	0.034		0.333	
	Caesarean section	−0.075 (−0.190–0.039)	0.202	0.081	
	Maternal smoking during pregnancy	−0.090 (−0.203–0.023)	0.129		0.190	
	Maternal anxiety during pregnancy	0.026 (−0.065–0.117)	0.579		0.036	
	Maternal gestational weight gain (kg·week^−1^)	−0.170 (−0.417–0.077)	0.183		0.055	
	Air pollution exposure during pregnancy (μg·m^−3^)	0.000 (−0.011–0.010)	0.977		0.013	
**Perinatal characteristics**	Maternal smoking during first year of age	−0.146 (−0.316–0.025)	0.103	0.40	0.164	1.07
	Day care attendance during first year of age	0.063 (−0.067–0.194)	0.344		0.036	
	Family pet ownership during first year of age	0.030 (−0.041–0.100)	0.412		0.029	
	Maternal anxiety during first year of age	0.029 (−0.041–0.099)	0.422		0.020	
	Air pollution during first year of age (μg·m^−3^)	−0.002 (−0.016–0.011)	0.726		0.020	
	Breastfeeding during first 6 months	−0.065 (−0.187–0.056)	0.300		0.094	
	Early secondhand smoke exposure	−0.024 (−0.112–0.063)	0.587		0.036	
**Early-childhood characteristics**	Secondhand smoke exposure during age 1–8 years	−0.063 (−0.134–0.008)	0.088	1.21	0.095	2.17
	Air pollution during 1–7 years of age (μg·m^−3^)	−0.001 (−0.010–0.008)	0.807		0.019	
	Lean mass at age 9 years (sd score)	0.035 (0.012–0.058)	0.004		0.309	
	Fat mass at age 9 years (sd score)	−0.006 (−0.024–0.013)	0.566		0.033	
	Current asthma at 7.5 years	−0.239 (−0.339–−0.139)	2×10^−5^		0.695	
	Skin prick test at 7.5 years	0.064 (−0.045–0.173)	0.257		0.059	
**Adolescence characteristics**	Smoking status at 14 years	−0.031 (−0.120–0.058)	0.495	0.09	0.042	2.17
	Age at peak height velocity in puberty (years)	0.014 (−0.011–0.038)	0.279		0.031	
	Peak height velocity in puberty (cm·year^−1^)	−0.010 (−0.032–0.011)	0.351		0.021	

RI: relative importance (proportion of explained variation in lung function attributed to each variable, averaging over all its possible orderings among characteristics in same stage); FEF_25–75%_: forced expiratory flow at 25–75% of forced vital capacity. ^#^: adjusted for all variables in same stage in addition to characteristics from previous stages that yield p-value ≤0.10; ^¶^: for variables in the corresponding stage; ^+^: total R^2^ for retained variables (with p≤0.10) from previous stages and corresponding stage.

There was little evidence for associations between postnatal characteristics and lung function outcomes, and for associations between adolescence characteristics and FEV_1_/FVC or FEF_25–75%_.

### Relative importance of factors in lung function models

After adjusting for sex, age and height, the proportions of remaining variance in lung function parameters explained by studied characteristics (R^2^ of sd score models) were 10.8%, 6.7%, 3.5% and 2.4% for FVC, FEV_1_, FEV_1_/FVC and FEF_25–75%_, respectively.

**FIGURE 4:**
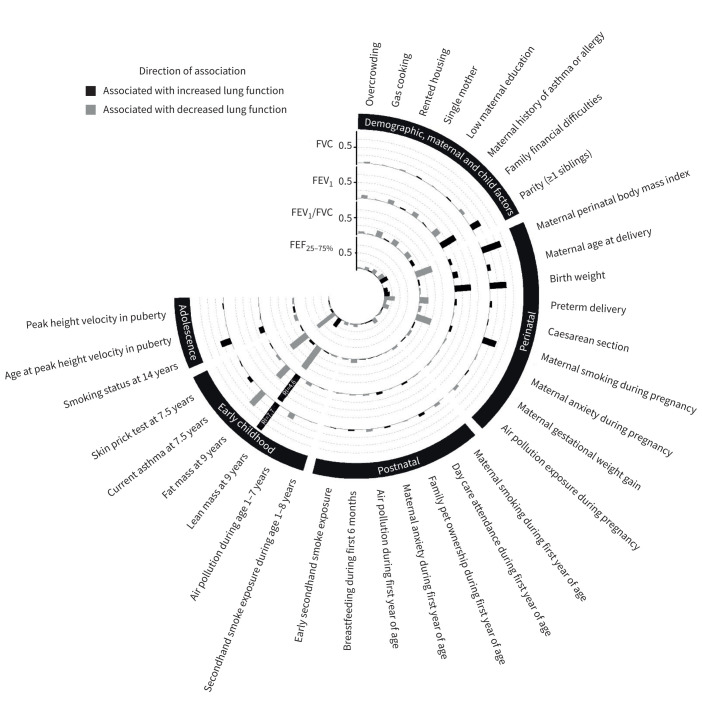
Circular plot of characteristics’ relative importance (RI) on lung function parameters at age 24 years. Associations with higher and lower lung function are highlighted in black and grey, respectively. Bar height represents levels of RI, expressed in %, except for characteristics whose RI was >1%, where exact RI values are displayed on their corresponding bars. FVC: forced vital capacity; FEV_1_: forced expiratory volume in 1 s; FEF_25–75%_: forced expiratory flow at 25–75% of FVC.

Figure 4 presents the RI of characteristics clustered by stage of life course for each spirometric parameter. Perinatal and early-childhood characteristics had the largest contributions to variations of all lung function parameters compared with other stages. For FVC, maternal perinatal BMI, birthweight, LM and FM at age 9 years had RI of 0.6%, 0.5%, 7.7% and 0.6%, respectively. For FEV_1_, parity (RI=0.5%), birthweight (RI=0.5%), LM (RI=4.6%) and FM (RI=0.5%) were the most important influences. For FEV_1_/FVC, maternal perinatal BMI (RI=0.5%), maternal smoking during pregnancy (RI=0.5%), LM (RI=0.8%) and asthma at age 7.5 years (RI=0.6%) had the greatest relative importance among studied characteristics. Asthma had the most important influence (RI=0.7%) on FEF_25–75%_ (tables 2–5).

Similar results for the associations and RI with lung function were obtained when restricting our analyses to only participants with measured (non-imputed) lung function at age 24 years (supplementary figures S2 and S3, supplementary tables S6–S9).

## Discussion

### Main findings

This large population-based birth cohort study investigated the associations of sociodemographic, environmental, lifestyle and physiological characteristics from prenatal to adolescence with lung function at age 24 years (around its physiological maximum) and derived the RI of each of these characteristics. With information on many exposures, our study showed that influences of perinatal and early-childhood characteristics were relatively larger than those of demographic, postnatal and adolescence characteristics. However, all influences were modest, *i.e.* the most influential characteristic, childhood LM, explained not more than 7.7% of the variation in lung function at age 24 years. Our study highlighted the RI of maternal perinatal BMI, birthweight, body composition in childhood, childhood asthma, socioeconomic status (as captured by self-reported financial difficulties and lower maternal education) and birth order on four major lung function parameters (FVC, FEV_1_, FEV_1_/FVC and FEF_25–75%_). Although exposure to air pollution (source-specific particulate matter with diameter ≤10 µm) during early childhood was associated with reduced lung volumes, we showed that it had much less influence on maximally attained levels of FVC and FEV_1_ compared to other characteristics such as birthweight and childhood body composition.

### Findings in the context of the literature

Our findings are in-line with the well-established evidence suggesting general primary roles of early-life exposures on adult lung function [12, 17, 28]. It had been shown that increased childhood BMI is associated with higher lung volume and airflow limitation in adolescents aged 15 years [30, 46]. By partitioning BMI into LM and FM, our study showed that higher LM and lower FM at age 9 years (both of which are likely to track throughout childhood) were associated with higher FVC and FEV_1_. These associations are described in another report from this study population looking at lung function at age 15 years [24]. Importantly our analysis suggests that of all the studied characteristics, LM has the largest influence on both FVC and FEV_1_. Moreover, we found that higher LM at age 9 years was associated with lower FEV_1_/FVC at age 24 years, which is likely to be attributable to LM having a higher influence on FVC than on FEV_1_. A similar finding, with a wider confidence interval, has been reported in a previous study [24] with FEV_1_/FVC at age 15 years. Our present study provides more evidence for such an association.

Previous studies provided strong and consistent evidence of an association of lower birthweight with adult restrictive lung function impairment, with weaker evidence for airflow obstruction [47]. Our study supports this, with larger RI for FVC than for FEV_1_/FVC (which was barely influenced by birthweight).

As might have been anticipated, having asthma by the age 7 years had a greater influence on FEV_1_/FVC and mid-expiratory flow than on lung volumes. Similar associations were reported with lung function in adolescence [10, 48].

A previous study showed that poverty is associated with lower lung function in adolescence [49]. Our study supports this, showing that children raised in families reporting family financial difficulties and with maternal lower education had lower lung function in early adulthood. This association played a bigger role in FEV_1_ reduction and airflow limitation than in FVC reduction.

Some of our findings are more difficult to interpret and explain. For example, having siblings was associated with increased lung function. Similar findings were previously reported for lung function in childhood [50, 51] with no adequate explanation of the mechanism of the association. Increased number of siblings has previously been shown to be inversely associated with asthma and hay fever at age 7 years, but this association did not persist after adjustment to the household size [52]. Our results for crude associations showed no association of having siblings with lung function in early adulthood, but this association appeared when the model was adjusted for the other demographic characteristics, including overcrowded household. Given that second-born babies tend to have higher birthweights compared with first-born babies [53], the association between parity and lung function might be due to differences in birthweight (weight at birth was positively associated with higher lung function). In a secondary analysis, we adjusted this association for birthweight and the results were only slightly attenuated. However, this secondary analysis might be liable to a collider bias induced by unmeasured common risk factors of birthweight and lung function [54].

Collider bias, residual confounding effects or a combination of both might also be a plausible explanation for the association between maternal smoking during pregnancy and higher FVC. We found clear evidence of detrimental effects of maternal smoking during pregnancy on FEV_1_/FVC, suggesting possible dysanapsis of lung growth, *i.e.* a physiological incongruence between the growth of the lung parenchyma and the calibre of the airways [55]. The association of smoking at age 14 years with increased FVC could be due to a selection bias, *e.g.* adolescents with larger lung volume might be more likely to initiate smoking. Because smokers were defined as those who have ever smoked at least one cigarette, this result does not account for the amount of smoking. Studying sub-categories of smoking might reveal more on the association between smoking in adolescence and lung function in early adulthood.

Higher maternal perinatal BMI was associated with reduced FEV_1_/FVC but increased FVC, suggesting that children of thinner mothers tended to have worse lung volumes. This may be a consequence of poor maternal perinatal nutrition and/or of poor childhood feeding for children of thinner mothers [56].

Early-life exposure to higher air pollution (source-specific particulate matter with diameter ≤10 µm) is believed to have an impact on developing lungs [57]. Our findings suggested less importance of the early-life exposure to air pollution compared with other childhood characteristics such as LM and FM.

### Implications of our study

There has long been interest in the relationship of persistent low lung function from early life with chronic pulmonary disease in later life but the importance of modifiable early-life characteristics on lung function has been unclear. Our study addressed roles of early-life characteristics, provided evidence for their association and quantified their RI on lung function in early adulthood, around the timing of its physiological maximum. This is relevant for a better understanding of lung function growth and factors likely to contribute to lower maximal lung function attainment. Our study suggests that the association of early-life risk factors, *e.g.* birthweight and childhood asthma, with impaired lung function in late adulthood [5, 8, 12] is likely related to their association with maximally attained lung function, and not solely due to their impacts on lung function decline [9].

Because various characteristics may influence, to a different extent, different lung function parameters, our assessment for RI of these characteristics can be beneficial in identifying the major determinants of restrictive and obstructive lung patterns. Our findings, together with earlier work showing evidence of lung function tracking throughout the life course, can help prioritise public health policies directed at children that target risk factors of low lung function in later life.

### Strengths and limitations

This study offers insights into the roles and relative importance of many early-life events on lung function at age 24 years, with all these characteristics simultaneously investigated. Given that many of these characteristics are clustered [58], studies investigating only a subset of them are liable to be at risk of confounding. Our study used a wide range of characteristics with measurements covering prenatal stage through to early adulthood, with a single large (n=7545) population-based birth cohort study (ALSPAC) and therefore provides a more comprehensive analysis across the life course. Inevitably some data were missing, but we have used state-of-the-art multiple imputation approaches to impute missing data, thus ensuring we were able to use all the information available to increase power and minimise bias related to selective loss to follow-up. We repeated our analyses using only participants with measured (non-imputed) lung function (n=2800). The results confirmed our findings obtained using the imputation approaches.

We used post-bronchodilator lung function parameters because they better represent the maximal lung function attained than their corresponding pre-bronchodilator values. The latter are not optimal when the study sample includes asthmatic patients because lung function measurement may be affected by reversible airway limitation.

Despite adjustments for a wide range of relevant characteristics, this study, like all observational studies, is still liable to residual confounding by unmeasured characteristics such as diet and physical activity that were only available for a small number of participants in our cohort. Furthermore, mutual adjustments of characteristics in the life-course stage might induce collider bias *via* such unmeasured confounders, although we believe that our extensive adjustments for potential confounding minimised the effects of such a bias. There is some evidence that men may reach maximal lung function later than women and we cannot rule out that this could have a small effect on our findings. Identification of the pathways through which characteristics affect lung function was beyond our remit. Our study adjusted only for events that are potential confounders, *i.e.* that occur earlier or at the time of exposure. However, mediation is a possible mechanism whereby earlier characteristics may influence lung function through factors that occur later, *e.g.* childhood characteristics might be mediators of perinatal characteristics.

### Conclusions

Beside well-known variables included in lung function equations (sex and height), our study provides evidence for associations of perinatal and childhood characteristics with early-adult lung function and quantifies their relative importance. Birthweight, having siblings, LM and FM at age 9 years were the most important influences on early-adult FVC and FEV_1_. Maternal perinatal BMI, smoking during pregnancy, preterm delivery, impaired childhood respiratory health and increased LM at age 9 years were associated with lower FEV_1_/FVC at age 24 years, with the largest detrimental effect from childhood asthma and LM. Childhood asthma, low LM and preterm delivery played the largest roles in low FEF_25–75%_.

Our findings highlight the importance of early-life characteristics in lung function and suggest public health polices targeting modifiable risk factors in childhood may improve maximally attained lung function and minimise poor respiratory health in later life.

## Supplementary material

10.1183/13993003.01316-2020.Supp1 **Please note:** supplementary material is not edited by the Editorial Office, and is uploaded as it has been supplied by the author.
Supplementary material ERJ-01316-2020.Supplement

## Shareable PDF

10.1183/13993003.01316-2020.Shareable1 This one-page PDF can be shared freely online.
Shareable PDF ERJ-01316-2020.Shareable
