# Supplementary material for: Early-life and health behaviour influences on lung function in early adulthood
Source: Eur Respir J. 2023 Mar 2;61(3):2001316. doi: 10.1183/13993003.01316-2020 (PMC9978163; doi:10.1183/13993003.01316-2020)
Supplement: Supplementary file 1 [file ERJ-01316-2020.Supplement.pdf]

Early-life and health behaviour influences on lung function in early-adulthood

Online Data Supplement

METHODS

Participants and data collection

ALSPAC initially recruited 14,541 pregnant women resident in Avon, UK with expected delivery dates between April 1, 1991 and December 31. This initial number of pregnancies, known as core sample, included the mothers enrolled in the ALSPAC study and had either returned at least one questionnaire or attended a 'Children in Focus' research clinic by 19th July 1999. These initial pregnancies had a total of 14,676 fetuses, resulting in 14,062 live births and 13,988 children who were alive at age one-year. When the oldest children were approximately seven years of age, an attempt was made to bolster the initial sample with eligible cases who had failed to join the study originally. As a result, there are extra data available when considering variables collected from the age of seven years onwards. The number of new pregnancies, not in the core sample, known as phases II and III enrolments, is 706 (452 and 254 recruited during Phases II and III respectively), resulting in an additional 713 children being enrolled. The phases of enrolment are described in more detail in the cohort profile paper (1). Therefore, the total sample size for which the ALSPAC data collected after the age of seven years is therefore 15,247 pregnancies, resulting in 15,458 fetuses with 14,775 live births and 14,701 alive children at one-year of age.

We restricted our study (N = 7,545) to the core sample participants who have lung function measured at least once, after excluding quadruplets, triplets and one random child of each twin births. The study was approved by the ALSPAC Ethics and Law Committee and local research ethics committees. Informed consent for the use of data collected via questionnaires and clinics was obtained from participants following the recommendations of the ALSPAC Ethics and Law Committee at the time.

Data were collected from several sources: self-administered questionnaires sent to mothers at approximately annual intervals from 6 to 198 months (16½ years); annual physical examinations carried out during research clinics from age 7 to 13 years and at 15, 17 and 24 years. Study data were collected and managed using REDCap electronic data capture tools(2, 3) hosted at University of Bristol. The ALSPAC study website contains details of all data through a fully searchable data dictionary that is available on the following Web page:

http://www.bris.ac.uk/alspac/researchers/data-access/data-dictionary/

Investigated characteristics

Factors were categorized according to their nature and timing: (1) demographic, maternal and child characteristics that do not change over time and/or were measured before birth, e.g. gas cooking, maternal asthma or allergy, family financial difficulties; (2) perinatal characteristics, e.g. birthweight, maternal smoking during pregnancy; (3) postnatal characteristics, e.g. maternal smoking during first year of age, air pollution exposure during first year of age; (4) early-childhood, e.g. exposure to second-hand smoking during age 1-8 years, air pollution exposure during age 1-7 years; lean and fat mass at age 9 years, current

asthma at age 7.5 years (5) adolescence characteristics, e.g. smoking status at age 14 years, pubertal age. Figure 1 shows an overview of investigated characteristics and a detailed description is provided in Table 1.

Lean mass and fat mass residuals

To adjust for differences in fat mass between females and males, and to adjust for height, the measures of fat mass and lean mass included in the analyses were calculated as the residuals from a linear regression of each on gender, height, and height squared. The standard deviation of fat mass residuals was 4.35 kg, approximately double that of lean mass (1.71 kg) residuals. We divided the fat mass residuals by two in subsequent analyses, so that regression coefficients for fat mass, and lean mass that were of similar size reflected associations of similar strength (4).

Statistical analysis

Dealing with missing data:

To assess whether missing values of lung function at age 24 years (outcomes) can plausibly be imputed using information from earlier lung function measurements, linear correlation coefficients between SD-scores of lung function measured at different ages were examined (Table 2). A layout of missing data of investigated characteristics and lung function outcomes at ages 8, 15 and 24 years was depicted in Figure S1.

Among the study population ($N = 7{,}545$), there were small amounts of missing data for all stages of factors (Figure S1). This varied from none, e.g. for pre-term delivery and maternal age at delivery, to 38%, for smoking status at age 14 years. The skin prick test (SPT) was

performed for only a selected random sub-sample. Therefore, the amount of its missing data was relatively high, 27%.

To increase power and minimize selection bias, multiple imputation by chained equations was performed to impute missing data among our study population (5). In our imputation models, we included all lung function measures at ages 8, 15 and 24 years, exposures, potential confounders, and additional variables that might be predictive of missingness or of the missing values themselves. These included all characteristics of interest as presented in Table 1, smoking status at ages 16, 18, 20, 22 and 23 years, current asthma at ages 9, 11, 13, 14 and 15, immunoglobulin-E blood test and maternal ever caesarean section delivery. We generated 20 imputed datasets using 10 cycles of regression switching (5). These datasets were then used for the main analyses. Since estimates of associations given by the regression models are derived to be normally distributed, we aggregated the findings across the imputed datasets using the Rubin's rules (6) and obtained 95% confidence intervals for characteristics' association by using the pooled means and pooled standard errors of estimated coefficients. The Rubin's rules produced average of individual coefficients and total average of between-imputation and within-imputation variances as the combined estimates of size of associations and their variances respectively.

Relative importance (RI) of influences on each lung function outcome were assessed using the Lindeman, Merenda, and Gold (LMG) method (7). The LMG analyses the explained variance, R^2, of a considered model and estimates the incremental R^2, defined as partial contribution to the total R^2, attributed to the characteristic of interest. The incremental R^2

for each characteristic might be influenced by the order in which its variable was entered in a model, particularly when correlations among variables exist, i.e. it is larger when the variable entered first and lower when entered last. The LMG derives RI for each characteristic using its incremental R^2 by averaging over all possible orderings among the set of characteristics in the same stage as the characteristic of interest. Thus, the relative importance of factors included in a model are normalized to sum up to its R^2. The procedures for calculating RI were implemented using the `relaimpo' R package (8).

RESULTS:

Table S1 reports characteristics of the study population using the observed and multiple imputed datasets. The summary statistics for most characteristics were similar in imputed and observed data due to their large proportions of observed data (≥ 90%) and the large size of study population ($N = 7{,}545$). For characteristics with lower proportions of observed data, including smoking status at age 14 years (62%), current asthma at age 7.5 years (73%), skin prick test at age 7.5 years (73%), air pollution during 1-7 years of age (81%) and second-hand smoke exposure during age 1-8 years, the differences in summary statistics between observed and imputed data were small, −0.6%, −2.3%, −0.01%, 0.06 $\mu g/m^3$, 1.7% respectively. Lung function, age and height distributions at 24 years clinic were similar in observed and imputed data, see Table S2. The medians and interquartile ranges (IQRs) of height and age were identical in both observed and imputed datasets.

We have reported the crude associations of the investigated characteristics with lung function measurements, only adjusted by sex, age and height, in Tables S10 – S13.

References

1. Boyd A, Golding J, Macleod J, Lawlor DA, Fraser A, Henderson J, Molloy L, Ness A, Ring S, Davey Smith G. Cohort Profile: the 'children of the 90s'--the index offspring of the Avon Longitudinal Study of Parents and Children. *Int J Epidemiol* 2013; 42: 111-127.
2. Harris PA, Taylor R, Thielke R, Payne J, Gonzalez N, Conde JG. Research electronic data capture (REDCap)--a metadata-driven methodology and workflow process for providing translational research informatics support. *J Biomed Inform* 2009; 42: 377-381.
3. Harris PA, Taylor R, Minor BL, Elliott V, Fernandez M, O'Neal L, McLeod L, Delacqua G, Delacqua F, Kirby J, Duda SN, Consortium RE. The REDCap consortium: Building an international community of software platform partners. *J Biomed Inform* 2019; 95: 103208.
4. Granell R, Henderson AJ, Evans DM, Smith GD, Ness AR, Lewis S, Palmer TM, Sterne JA. Effects of BMI, fat mass, and lean mass on asthma in childhood: a Mendelian randomization study. *PLoS Med* 2014; 11: e1001669.
5. Azur MJ, Stuart EA, Frangakis C, Leaf PJ. Multiple imputation by chained equations: what is it and how does it work? *Int J Methods Psychiatr Res* 2011; 20: 40-49.
6. Rubin DB. Multiple imputation after 18+ years. *J Am Stat Assoc* 1996; 91: 473-489.
7. Sawrey WL. Introduction to Bivariate and Multivariate-Analysis - Lindeman,Rh, Merenda,Pf, Gold,Rz. *Contemp Psychol* 1981; 26: 374-374.
8. Gromping U. Relative importance for linear regression in R: The package relaimpo. *J Stat Softw* 2006; 17.

TABLES

Table S1. Characteristics of study population using the observed and imputed datasets.

Stage	Factor	Observed data		Imputed data (N = 7545)
		N	% or Median	% or Median
Demographic, maternal and child characteristics	Overcrowding	7214	20.57	20.8
	Gas cooking	7222	53.96	53.95
	Rented housing	7020	14.09	14.98
	Single Mother	7356	18.28	18.54
	Low maternal education*	7225	57.49	57.99
	Maternal history of asthma or allergy	7110	47.33	47.27
	Family financial difficulties	7423	3.37	3.43
	Parity (>= 1 siblings)	7250	53.7	53.65
Perinatal characteristics	Maternal perinatal body mass index (Kg/m^2) †	6747	22.18	22.27
	Maternal age at delivery > 28 years (the median)	7528	53.21	53.21
	Birthweight (Kg) †	7437	3.44	3.44
	Pre-term delivery	7528	5.37	5.38
	Caesarean section	7357	10.51	10.5
	Maternal smoking during pregnancy	7146	20.86	21.05
	Maternal anxiety during pregnancy	6593	30.15	30.42
	Maternal gestational weight gain (Kg/week) †	6906	0.43	0.42
	Air pollution exposure during pregnancy (μg/m^3) †	6983	32.72	32.7
Postnatal characteristics	Maternal smoking during first year of age	6929	18.82	20.05
	Day care attendance during first year of age	6879	6.72	6.63
	Family pet ownership during first year of age	6938	69.07	69.27
	Maternal anxiety during first year of age	6893	21.44	21.93
	Air pollution during first year of age (μg/m^3) †	6932	31.38	31.38
	Breastfeeding during first 6 months	6978	81.76	81.23
	Early second-hand smoke exposure	7031	33.15	34.25
Early-Childhood characteristics	Second-hand smoke exposure during age 1-8 y	6333	62.92	61.14
	Air pollution during 1-7 years of age (μg/m^3) †	6140	73.14	73.07
	Lean mass at age 9 years (kg) †	5707	-0.12	-0.1
	Fat mass at age 9 years (kg/2) †	5707	-0.47	-0.37
	Current asthma at 7.5 years	5533	14.19	16.52
	Skin Prick Test at 7.5 years	5522	21.06	21.09
Adoles.	Smoking status at 14 years	4679	24.96	25.68
	Age at peak height velocity in puberty (years) †	7240	12.48	12.48
	Peak height velocity in puberty (cm/year) †	7240	8.88	8.89

Abbreviations: Adoles. = adolescence; Kg = kilogram; m = metre; μg = microgram; cm = centimetre.

*Educated to the General Certificate of Education level (school-leaving certificate) or lower, see **Error! Reference source not found.**.

†Continuous variables where medians were calculated.

Table S2. Comparisons of the characteristics of study population with the original ALSPAC population.

Stage	Factor	Study population (N = 7,545)		Original ALSPAC population (N= 13,798)	
		N	% or Median	N	% or Median
Demographic, maternal and child characteristics	Overcrowding	7214	20.57	12645	27.01
	Gas cooking	7222	53.96	12731	52.63
	Rented housing	7020	14.09	12202	22.68
	Single Mother	7356	18.28	12980	24.38
	Low maternal education*	7225	57.49	12261	64.62
	Maternal history of asthma or allergy	7110	47.33	12047	45.56
	Family financial difficulties	7423	3.37	12807	5.36
	Parity (>= 1 siblings)	7250	53.7	12765	55.22
Perinatal characteristics	Maternal perinatal body mass index (Kg/m^2) †	6747	22.18	11374	22.18
	Maternal age at delivery > 28 years (the median)	7528	53.21	13798	45.14
	Birthweight (Kg) †	7437	3.44	13622	3.42
	Pre-term delivery	7528	5.37	13798	5.55
	Caesarean section	7357	10.51	8116	17.58
	Maternal smoking during pregnancy	7146	20.86	12171	29.52
	Maternal anxiety during pregnancy	6593	30.15	11120	34.07
	Maternal gestational weight gain (Kg/week) †	6906	0.43	12438	0.42
	Air pollution exposure during pregnancy ($\mu g/m^3$) †	6983	32.72	12638	32.67
Postnatal characteristics	Maternal smoking during first year of age	6929	18.82	11073	24.2
	Day care attendance during first year of age	6879	6.72	10825	6
	Family pet ownership during first year of age	6938	69.07	10941	69.42
	Maternal anxiety during first year of age	6893	21.44	11023	22.42
	Air pollution during first year of age ($\mu g/m^3$) †	6932	31.38	12493	31.36
	Breastfeeding during first 6 months	6978	81.76	11196	75.8
	Early second-hand smoke exposure	7031	33.15	11328	38.06
Early-Childhood characteristics	Second-hand smoke exposure during age 1-8 y	6333	62.92	9804	72.23
	Air pollution during 1-7 years of age ($\mu g/m^3$) †	6140	73.14	10896	73.12
	Lean mass at age 9 years (kg) †	5707	-0.12	6339	-0.11
	Fat mass at age 9 years (kg/2) †	5707	-0.47	6339	-0.46
	Current asthma at 7.5 years	5533	14.19	7218	14.13
	Skin Prick Test at 7.5 years	5522	21.06	6438	20.69
Adoles.	Smoking status at 14 years	4679	24.96	5586	25.67
	Age at peak height velocity in puberty (years) †	7240	12.48	9032	12.5
	Peak height velocity in puberty (cm/year) †	7240	8.88	9032	8.91

Considered ALSPAC population = Singleton and one twin birth alive at age 1 year of the Avon Longitudinal Study of Parents and Children.

Abbreviations: Adoles. = adolescence; Kg = kilogram; m = metre; μg = microgram; cm = centimetre.

*Educated to the General Certificate of Education level (school-leaving certificate) or lower, see **Error! Reference source not found.**.

†Continuous variables where medians were calculated.

Table S3. Comparisons of the characteristics of participants in the study population with and without the lung function measurements at age 24 years.

Stage	Factor	With Lung function measurements at age 24 years (N= 2,800)		Without lung function measurements at age 24 years (N= 4,745)	
		N	% or Median	N	% or Median
Demographic, maternal and child characteristics	Overcrowding	2692	17.27	4522	22.53
	Gas cooking	2690	54.68	4532	53.53
	Rented housing	2624	10.02	4396	16.52
	Single Mother	2745	15.63	4611	19.87
	Low maternal education*	2711	50.02	4514	61.98
	Maternal history of asthma or allergy	2660	49.32	4450	46.13
	Family financial difficulties	2759	2.79	4664	3.71
	Parity (>= 1 siblings)	2710	53.1	4540	54.05
Perinatal characteristics	Maternal perinatal body mass index (Kg/m^2) †	2543	21.97	4204	22.36
	Maternal age at delivery > 28 years (the median)	2794	58.2	4734	50.27
	Birthweight (Kg) †	2758	3.43	4679	3.45
	Pre-term delivery	2794	5.05	4734	5.56
	Caesarean section	2740	10.88	4617	10.29
	Maternal smoking during pregnancy	2666	16.69	4480	23.35
	Maternal anxiety during pregnancy	2465	28.72	4128	31.01
	Maternal gestational weight gain (Kg/week) †	2558	0.43	4348	0.42
	Air pollution exposure during pregnancy (μg/m^3) †	2591	32.76	4392	32.7
Postnatal characteristics	Maternal smoking during first year of age	2599	15.04	4330	21.09
	Day care attendance during first year of age	2585	7.58	4294	6.19
	Family pet ownership during first year of age	2605	68.25	4333	69.56
	Maternal anxiety during first year of age	2587	20.6	4306	21.95
	Air pollution during first year of age (μg/m^3) †	2561	31.41	4371	31.35
	Breastfeeding during first 6 months	2625	85.56	4353	79.46
	Early second-hand smoke exposure	2637	28.97	4394	35.66
Early-Childhood characteristics	Second-hand smoke exposure during age 1-8 y	2378	55.38	3955	67.46
	Air pollution during 1-7 years of age (μg/m^3) †	2219	73.09	3921	73.16
	Lean mass at age 9 years (kg) †	2132	-0.14	3575	-0.11
	Fat mass at age 9 years (kg/2) †	2132	-0.5	3575	-0.46
	Current asthma at 7.5 years	2119	13.17	3414	14.82
	Skin Prick Test at 7.5 years	2068	20.94	3454	21.13
Adoles.	Smoking status at 14 years	2128	23.03	2551	26.58
	Age at peak height velocity in puberty (years) †	2693	12.24	4547	12.69
	Peak height velocity in puberty (cm/year) †	2693	8.6	4547	9.06

Abbreviations: Adoles. = adolescence; Kg = kilogram; m = metre; μg = microgram; cm = centimetre.

*Educated to the General Certificate of Education level (school-leaving certificate) or lower, see **Error! Reference source not found.**.

†Continuous variables where medians were calculated.

Table S4. Summary statistics of lung function outcomes at age 24 years for the study population using the observed and imputed datasets.

Variable	Observed data		Imputed data (N = 7545)
	N	Median (IQR)	Median (IQR)
Age (years)	3391	24.4 (23.9 to 25.1)	24.5 (23.9 to 25.1)
Height at lung function clinic (metres)	3371	1.7 (1.6 to 1.8)	1.7 (1.6 to 1.8)
Forced vital capacity, FVC (L)	2800	4.3 (3.7 to 5.2)	4.6 (3.9 to 5.5)
Forced expiratory volume in one second, FEV_1 (L)	2800	3.7 (3.2 to 4.4)	3.9 (3.3 to 4.6)
FEV_1/FVC (%)	2800	85.9 (82.0 to 89.5)	85.4 (81.0 to 89.4)
Forced expiratory flow, midexpiratory phase (L/s)	2800	4.1 (3.4 to 4.8)	4.2 (3.5 to 5.0)

Abbreviations: IQR= interquartile range ; L=litre ; s=second

Table S5. Pairwise linear correlation coefficients between SD scores (adjusted for sex, age and height) of lung function measurements at different timepoints.

	FVC		FEV_1		FEF_{25-75}	
	**15 years**	**24 years**	**15 years**	**24 years**	**15 years**	**24 years**
**8 years**	0.399	0.504	0.372	0.508	0.424	0.513
**15 years**		0.475		0.455		0.484

Table S6. Adjusted association and relative importance of early-life characteristics with SD scores (adjusted for sex, age and height) of FVC measurements (non-imputed) at age 24 years (N=2800).

Stage	Factor	Adjusted* difference in SD scores of FVC (95% CI)	P-value	Inc. R^2 (%)	RI(%)	Retained R^2
Demographic, maternal and child characteristics	Overcrowding	-0.084 (-0.191 to 0.023)	0.126	0.64	0.078	0.40
	Gas cooking	0.016 (-0.060 to 0.091)	0.683		0.011	
	Rented housing	-0.080 (-0.225 to 0.065)	0.280		0.073	
	Single Mother	0.077 (-0.035 to 0.189)	0.178		0.031	
	Low maternal education	-0.004 (-0.080 to 0.073)	0.921		0.004	
	Maternal history of asthma or allergy	0.029 (-0.047 to 0.106)	0.448		0.020	
	Family financial difficulties	-0.098 (-0.329 to 0.132)	0.404		0.028	
	Parity (>= 1 siblings)	0.139 (0.062 to 0.217)	4×10^{-4}		0.398	
Perinatal characteristics	Maternal perinatal body mass index (Kg/m^2)	0.017 (0.006 to 0.028)	0.003	1.56	0.413	1.82
	Maternal age at delivery > 28 years (the median)	0.086 (0.008 to 0.164)	0.030		0.141	
	Birthweight (Kg)	0.163 (0.080 to 0.247)	1×10^{-4}		0.561	
	Pre-term delivery	0.144 (-0.046 to 0.334)	0.139		0.042	
	Caesarean section	0.019 (-0.103 to 0.141)	0.759		0.008	
	Maternal smoking during pregnancy	0.174 (0.070 to 0.277)	0.001		0.309	
	Maternal anxiety during pregnancy	-0.034 (-0.120 to 0.053)	0.449		0.015	
	Maternal gestational weight gain (Kg/week)	0.066 (-0.222 to 0.355)	0.653		0.025	
	Air pollution exposure during pregnancy (µg/m^3)	-0.007 (-0.019 to 0.005)	0.261		0.049	
Postnatal characteristics	Maternal smoking during first year of age	-0.096 (-0.263 to 0.071)	0.261	0.38	0.063	1.82
	Day care attendance during first year of age	0.107 (-0.039 to 0.252)	0.150		0.084	
	Family pet ownership during first year of age	-0.056 (-0.142 to 0.029)	0.197		0.073	
	Maternal anxiety during first year of age	0.001 (-0.092 to 0.095)	0.977		0.002	
	Air pollution during first year of age (µg/m^3)	-0.010 (-0.025 to 0.005)	0.200		0.059	
	Breastfeeding during first 6 months	0.085 (-0.027 to 0.196)	0.137		0.098	
	Early second-hand smoke exposure	-0.001 (-0.090 to 0.088)	0.978		0.005	
Early-Childhood characteristics	Second-hand smoke exposure during age 1-8 y	0.018 (-0.065 to 0.101)	0.669	7.93	0.014	9.69
	Air pollution during 1-7 years of age (µg/m^3)	-0.008 (-0.018 to 0.002)	0.098		0.131	
	Lean mass at age 9 years (SD-score)	0.178 (0.153 to 0.203)	1×10^{-16}		7.115	
	Fat mass at age 9 years (SD-score)	-0.056 (-0.076 to -0.037)	1×10^{-8}		0.621	
	Current asthma at 7.5 years	0.005 (-0.109 to 0.120)	0.927		0.014	
	Skin Prick Test at 7.5 years	0.047 (-0.067 to 0.161)	0.418		0.035	
Adoles.	Smoking status at 14 years	0.124 (0.032 to 0.216)	0.009	0.29	0.256	9.95
	Age at peak height velocity in puberty (years)	0.014 (-0.018 to 0.047)	0.387		0.021	
	Peak height velocity in puberty (cm/year)	0.007 (-0.021 to 0.035)	0.612		0.012	

Abbreviations: Adoles. = adolescence characteristics; FVC = forced vital capacity; Inc. R^2 = incremental R^2 for variables in the corresponding stage; RI = relative importance (proportion of explained variation in lung function attributed to each variable – averaging over all its possible orderings among characteristics in same stage); Retained R^2 = Total R^2 for retained variables (with P-value ≤ 0.10) from previous stages and corresponding stage, Kg = kilogram; m = metre; µg = microgram; cm = centimetre.

* Adjusted for all variables in same stage in addition to characteristics from previous stages that yield P-value ≤ 0.10.

Table S7. Adjusted association and relative importance of early-life characteristics with SD scores (adjusted for sex, age and height) of FEV_1 measurements (non-imputed) at age 24 years (N=2800).

Stage	Factor	Adjusted* difference in SD scores of FVC (95% CI)	P-value	Inc. R^2 (%)	RI(%)	Retained R^2
Demographic, maternal and child characteristics	Overcrowding	-0.088 (-0.194 to 0.019)	0.108	1.10	0.102	0.70
	Gas cooking	0.027 (-0.049 to 0.102)	0.485		0.029	
	Rented housing	-0.105 (-0.250 to 0.040)	0.157		0.137	
	Single Mother	0.088 (-0.024 to 0.200)	0.122		0.036	
	Low maternal education	-0.050 (-0.126 to 0.027)	0.204		0.084	
	Maternal history of asthma or allergy	0.020 (-0.056 to 0.097)	0.605		0.011	
	Family financial difficulties	-0.222 (-0.452 to 0.008)	0.058		0.143	
	Parity (>= 1 siblings)	0.165 (0.088 to 0.242)	3×10^{-5}		0.556	
Perinatal characteristics	Maternal perinatal body mass index (Kg/m^2)	0.004 (-0.007 to 0.016)	0.465	1.12	0.052	1.60
	Maternal age at delivery > 28 years (the median)	0.079 (0.001 to 0.156)	0.048		0.133	
	Birthweight (Kg)	0.186 (0.102 to 0.270)	1×10^{-5}		0.771	
	Pre-term delivery	0.041 (-0.149 to 0.232)	0.671		0.063	
	Caesarean section	-0.016 (-0.137 to 0.106)	0.799		0.006	
	Maternal smoking during pregnancy	0.084 (-0.020 to 0.188)	0.115		0.054	
	Maternal anxiety during pregnancy	-0.039 (-0.126 to 0.048)	0.382		0.027	
	Maternal gestational weight gain (Kg/week)	-0.047 (-0.337 to 0.242)	0.749		0.014	
	Air pollution exposure during pregnancy ($\mu g/m^3$)	-0.002 (-0.014 to 0.011)	0.801		0.004	
Postnatal characteristics	Maternal smoking during first year of age	-0.003 (-0.117 to 0.111)	0.957	0.11	0.003	1.60
	Day care attendance during first year of age	0.067 (-0.077 to 0.211)	0.360		0.034	
	Family pet ownership during first year of age	-0.013 (-0.097 to 0.071)	0.753		0.006	
	Maternal anxiety during first year of age	0.042 (-0.053 to 0.137)	0.389		0.031	
	Air pollution during first year of age ($\mu g/m^3$)	-0.005 (-0.020 to 0.009)	0.487		0.018	
	Breastfeeding during first 6 months	0.021 (-0.091 to 0.134)	0.709		0.010	
	Early second-hand smoke exposure	0.011 (-0.078 to 0.099)	0.811		0.003	
Early-Childhood characteristics	Second-hand smoke exposure during age 1-8 y	0.045 (-0.037 to 0.127)	0.286	5.81	0.057	7.28
	Air pollution during 1-7 years of age ($\mu g/m^3$)	-0.005 (-0.015 to 0.005)	0.330		0.047	
	Lean mass at age 9 years (SD-score)	0.150 (0.124 to 0.176)	1×10^{-16}		4.983	
	Fat mass at age 9 years (SD-score)	-0.052 (-0.071 to -0.032)	2×10^{-7}		0.573	
	Current asthma at 7.5 years	-0.101 (-0.220 to 0.017)	0.096		0.121	
	Skin Prick Test at 7.5 years	0.035 (-0.083 to 0.152)	0.564		0.024	
Adoles.	Smoking status at 14 years	0.072 (-0.024 to 0.169)	0.141	0.13	0.092	7.28
	Age at peak height velocity in puberty (years)	0.018 (-0.015 to 0.050)	0.286		0.032	
	Peak height velocity in puberty (cm/year)	-0.006 (-0.034 to 0.022)	0.683		0.006	

Abbreviations: Adoles. = adolescence characteristics; FEV_1 = forced expiratory volume in one second; Inc. R^2 = incremental R^2 for variables in the corresponding stage; RI = relative importance (proportion of explained variation in lung function attributed to each variable – averaging over all its possible orderings among characteristics in same stage); Retained R^2 = Total R^2 for retained variables (with P-value ≤ 0.10) from previous stages and corresponding stage, Kg = kilogram; m = metre; µg = microgram; cm = centimetre.

* Adjusted for all variables in same stage in addition to characteristics from previous stages that yield P-value ≤ 0.10.

Table S8. Adjusted association and relative importance of early-life characteristics with SD scores (adjusted for sex, age and height) of FEV_1/FVC measurements (non-imputed) at age 24 years (N=2800).

Stage	Factor	Adjusted* difference in SD scores of FVC (95% CI)	P-value	Inc. R^2 (%)	RI(%)	Retained R^2
Demographic, maternal and child characteristics	Overcrowding	-0.017 (-0.125 to 0.091)	0.755	0.46	0.015	0.33
	Gas cooking	0.006 (-0.071 to 0.083)	0.883		0.005	
	Rented housing	-0.047 (-0.187 to 0.092)	0.504		0.046	
	Single Mother	0.007 (-0.105 to 0.118)	0.908		0.008	
	Low maternal education	-0.080 (-0.157 to -0.004)	0.040		0.173	
	Maternal history of asthma or allergy	-0.018 (-0.094 to 0.058)	0.642		0.008	
	Family financial difficulties	-0.228 (-0.460 to 0.003)	0.053		0.159	
	Parity (>= 1 siblings)	0.047 (-0.030 to 0.124)	0.232		0.046	
Perinatal characteristics	Maternal perinatal body mass index (Kg/m^2)	-0.019 (-0.030 to -0.008)	0.001	1.23	0.396	1.31
	Maternal age at delivery > 28 years (the median)	-0.006 (-0.085 to 0.072)	0.875		0.001	
	Birthweight (Kg)	0.026 (-0.057 to 0.110)	0.538		0.056	
	Pre-term delivery	-0.220 (-0.411 to -0.029)	0.024		0.238	
	Caesarean section	-0.077 (-0.199 to 0.044)	0.210		0.090	
	Maternal smoking during pregnancy	-0.161 (-0.269 to -0.053)	0.003		0.340	
	Maternal anxiety during pregnancy	-0.007 (-0.097 to 0.083)	0.879		0.015	
	Maternal gestational weight gain (Kg/week)	-0.182 (-0.470 to 0.106)	0.215		0.032	
	Air pollution exposure during pregnancy ($\mu g/m^3$)	0.007 (-0.005 to 0.020)	0.248		0.057	
Postnatal characteristics	Maternal smoking during first year of age	-0.056 (-0.228 to 0.116)	0.523	0.22	0.020	1.31
	Day care attendance during first year of age	-0.059 (-0.204 to 0.087)	0.429		0.025	
	Family pet ownership during first year of age	0.042 (-0.041 to 0.125)	0.318		0.039	
	Maternal anxiety during first year of age	0.060 (-0.037 to 0.156)	0.225		0.058	
	Air pollution during first year of age ($\mu g/m^3$)	0.002 (-0.013 to 0.017)	0.803		0.003	
	Breastfeeding during first 6 months	-0.076 (-0.189 to 0.037)	0.189		0.071	
	Early second-hand smoke exposure	-0.004 (-0.094 to 0.086)	0.934		0.002	
Early-Childhood characteristics	Second-hand smoke exposure during age 1-8 y	-0.004 (-0.090 to 0.083)	0.930	1.34	0.006	2.55
	Air pollution during 1-7 years of age ($\mu g/m^3$)	0.004 (-0.006 to 0.014)	0.437		0.028	
	Lean mass at age 9 years (SD-score)	-0.052 (-0.078 to -0.025)	1×10^{-4}		0.707	
	Fat mass at age 9 years (SD-score)	0.001 (-0.021 to 0.023)	0.939		0.041	
	Current asthma at 7.5 years	-0.199 (-0.325 to -0.072)	0.003		0.536	
	Skin Prick Test at 7.5 years	0.007 (-0.101 to 0.114)	0.904		0.026	
Adoles.	Smoking status at 14 years	-0.087 (-0.194 to 0.019)	0.109	0.22	0.151	2.55
	Age at peak height velocity in puberty (years)	0.013 (-0.020 to 0.045)	0.453		0.024	
	Peak height velocity in puberty (cm/year)	-0.018 (-0.047 to 0.010)	0.210		0.049	

Abbreviations: Adoles. = adolescence characteristics; FEV_1 = forced expiratory volume in one second; FVC = forced vital capacity ; Inc. R^2 = incremental R^2 for variables in the corresponding stage; RI = relative importance (proportion of explained variation in lung function attributed to each variable – averaging over all its possible orderings among characteristics in same stage); Retained R^2 = Total R^2 for retained variables from previous stages and corresponding stage, Kg = kilogram; m = metre; µg = microgram; cm = centimetre.

* Adjusted for all variables in same stage in addition to characteristics from previous stages that yield P-value ≤ 0.10.

Table S9. Adjusted association and relative importance of early-life characteristics with SD scores (adjusted for sex, age and height) of FEF_{25-75} measurements (non-imputed) at age 24 years (N=2800).

Stage	Factor	Adjusted* difference in SD scores of FVC (95% CI)	P-value	Inc. R^2 (%)	RI(%)	Retained R^2
Demographic, maternal and child characteristics	Overcrowding	-0.016 (-0.124 to 0.093)	0.775	0.59	0.009	0.41
	Gas cooking	0.020 (-0.056 to 0.097)	0.607		0.016	
	Rented housing	-0.048 (-0.190 to 0.094)	0.508		0.042	
	Single Mother	0.031 (-0.081 to 0.143)	0.589		0.007	
	Low maternal education	-0.059 (-0.137 to 0.018)	0.131		0.099	
	Maternal history of asthma or allergy	-0.006 (-0.083 to 0.071)	0.879		0.003	
	Family financial difficulties	-0.267 (-0.497 to -0.037)	0.023		0.199	
	Parity (>= 1 siblings)	0.099 (0.022 to 0.176)	0.012		0.212	
Perinatal characteristics	Maternal perinatal body mass index (Kg/m^2)	0.000 (-0.012 to 0.011)	0.959	0.87	0.004	1.02
	Maternal age at delivery > 28 years (the median)	0.038 (-0.040 to 0.116)	0.342		0.039	
	Birthweight (Kg)	0.089 (0.005 to 0.173)	0.039		0.309	
	Pre-term delivery	-0.206 (-0.397 to -0.015)	0.034		0.296	
	Caesarean section	-0.073 (-0.194 to 0.048)	0.239		0.065	
	Maternal smoking during pregnancy	-0.081 (-0.186 to 0.024)	0.132		0.105	
	Maternal anxiety during pregnancy	0.014 (-0.074 to 0.103)	0.749		0.006	
	Maternal gestational weight gain (Kg/week)	-0.139 (-0.428 to 0.150)	0.347		0.021	
	Air pollution exposure during pregnancy ($\mu g/m^3$)	0.005 (-0.008 to 0.017)	0.441		0.027	
Postnatal characteristics	Maternal smoking during first year of age	-0.136 (-0.304 to 0.032)	0.112	0.25	0.099	1.02
	Day care attendance during first year of age	0.038 (-0.107 to 0.183)	0.606		0.012	
	Family pet ownership during first year of age	0.051 (-0.032 to 0.134)	0.231		0.054	
	Maternal anxiety during first year of age	0.052 (-0.046 to 0.151)	0.298		0.046	
	Air pollution during first year of age ($\mu g/m^3$)	0.001 (-0.014 to 0.016)	0.900		0.002	
	Breastfeeding during first 6 months	-0.051 (-0.163 to 0.061)	0.373		0.034	
	Early second-hand smoke exposure	-0.004 (-0.093 to 0.085)	0.930		0.002	
Early-Childhood characteristics	Second-hand smoke exposure during age 1-8 y	-0.020 (-0.107 to 0.067)	0.650	1.30	0.014	2.24
	Air pollution during 1-7 years of age ($\mu g/m^3$)	0.002 (-0.008 to 0.012)	0.690		0.007	
	Lean mass at age 9 years (SD-score)	0.047 (0.020 to 0.073)	0.001		0.509	
	Fat mass at age 9 years (SD-score)	-0.008 (-0.029 to 0.013)	0.448		0.024	
	Current asthma at 7.5 years	-0.243 (-0.362 to -0.124)	8×10^{-5}		0.716	
	Skin Prick Test at 7.5 years	0.053 (-0.051 to 0.158)	0.319		0.029	
Adoles	Smoking status at 14 years	-0.047 (-0.151 to 0.057)	0.381	0.09	0.051	2.24
	Age at peak height velocity in puberty (years)	0.013 (-0.020 to 0.046)	0.437		0.024	
	Peak height velocity in puberty (cm/year)	-0.009 (-0.037 to 0.019)	0.535		0.012	

Abbreviations: Adoles. = adolescence characteristics; FEF_{25-75} = forced expiratory flow, midexpiratory phase; Inc. R^2 = incremental R^2 for variables in the corresponding stage; RI = relative importance (proportion of explained variation in lung function attributed to each variable – averaging over all its possible orderings among characteristics in same stage); Retained R^2 = Total R^2 for retained variables (with P-value ≤ 0.10) from previous stages and corresponding stage, Kg = kilogram; m = metre; µg = microgram; cm = centimetre

* Adjusted for all variables in same stage in addition to characteristics from previous stages that yield P-value ≤ 0.10.

Table S10. Crude associations of early-life characteristics with SD scores of FVC (scores adjusted for sex, age and height) at age 24 years (N=7545).

Stage	Factor	Difference in SD scores of FVC (95% CI)	P-value
Demographic, maternal and child characteristics	Overcrowding	-0.034 (-0.114 to 0.046)	0.402
	Gas cooking	-0.009 (-0.077 to 0.060)	0.806
	Rented housing	-0.019 (-0.144 to 0.105)	0.762
	Single Mother	0.012 (-0.069 to 0.094)	0.769
	Low maternal education*	-0.024 (-0.083 to 0.034)	0.417
	Maternal history of asthma or allergy	0.027 (-0.041 to 0.095)	0.442
	Family financial difficulties	-0.127 (-0.377 to 0.123)	0.326
	Parity (>= 1 siblings)	0.102 (0.033 to 0.170)	0.005
Perinatal characteristics	Maternal perinatal body mass index (Kg/m^2)	0.023 (0.012 to 0.033)	0.000
	Maternal age at delivery > 28 years (the median)	0.073 (0.004 to 0.141)	0.043
	Birthweight (Kg)	0.146 (0.083 to 0.208)	0.000
	Pre-term delivery	-0.025 (-0.198 to 0.148)	0.778
	Caesarean section	0.020 (-0.103 to 0.142)	0.754
	Maternal smoking during pregnancy	0.136 (0.032 to 0.240)	0.015
	Maternal anxiety during pregnancy	-0.011 (-0.093 to 0.070)	0.786
	Maternal gestational weight gain (Kg/week)	0.040 (-0.199 to 0.280)	0.743
	Air pollution exposure during pregnancy ($\mu g/m^3$)	-0.009 (-0.021 to 0.002)	0.111
Postnatal characteristics	Maternal smoking during first year of age	0.065 (-0.038 to 0.168)	0.225
	Day care attendance during first year of age	0.074 (-0.058 to 0.206)	0.277
	Family pet ownership during first year of age	-0.025 (-0.092 to 0.041)	0.456
	Maternal anxiety during first year of age	0.049 (-0.041 to 0.139)	0.291
	Air pollution during first year of age ($\mu g/m^3$)	-0.010 (-0.022 to 0.003)	0.147
	Breastfeeding during first 6 months	0.002 (-0.095 to 0.099)	0.969
	Early second-hand smoke exposure	0.045 (-0.026 to 0.117)	0.220
Early-Childhood characteristics	Second-hand smoke exposure during age 1-8 y	0.050 (-0.011 to 0.111)	0.109
	Air pollution during 1-7 years of age ($\mu g/m^3$)	-0.011 (-0.020 to -0.002)	0.017
	Lean mass at age 9 years (kg) †	0.169 (0.151 to 0.188)	0.000
	Fat mass at age 9 years (kg/2) †	0.007 (-0.012 to 0.025)	0.475
	Current asthma at 7.5 years	0.092 (-0.029 to 0.214)	0.145
	Skin Prick Test at 7.5 years	0.008 (-0.089 to 0.105)	0.874
Adoles.	Smoking status at 14 years	0.163 (0.067 to 0.258)	0.002
	Age at peak height velocity in puberty (years)	-0.013 (-0.035 to 0.009)	0.248
	Peak height velocity in puberty (cm/year)	0.011 (-0.010 to 0.033)	0.290

Abbreviations: FVC = forced vital capacity; Adoles. = adolescence; Kg = kilogram; m = metre; µg = microgram; cm = centimetre.

*Educated to the General Certificate of Education level (school-leaving certificate) or lower, see **Error! Reference source not found.**.

Table S11. Crude associations of early-life characteristics with SD scores of FEV_1 (scores adjusted for sex, age and height) at age 24 years (N=7545).

Stage	Factor	Difference in SD scores of FEV_1 (95% CI)	P-value
Demographic, maternal and child characteristics	Overcrowding	-0.074 (-0.157 to 0.009)	0.084
	Gas cooking	-0.008 (-0.076 to 0.061)	0.828
	Rented housing	-0.103 (-0.235 to 0.029)	0.136
	Single Mother	-0.018 (-0.108 to 0.072)	0.695
	Low maternal education*	-0.081 (-0.147 to -0.016)	0.018
	Maternal history of asthma or allergy	0.021 (-0.050 to 0.092)	0.564
	Family financial difficulties	-0.252 (-0.461 to -0.042)	0.023
	Parity (>= 1 siblings)	0.130 (0.060 to 0.200)	0.001
Perinatal characteristics	Maternal perinatal body mass index (Kg/m^2)	0.009 (0.000 to 0.018)	0.068
	Maternal age at delivery > 28 years (the median)	0.111 (0.051 to 0.171)	0.000
	Birthweight (Kg)	0.162 (0.103 to 0.221)	0.000
	Pre-term delivery	-0.169 (-0.324 to -0.014)	0.037
	Caesarean section	-0.043 (-0.161 to 0.075)	0.479
	Maternal smoking during pregnancy	0.008 (-0.102 to 0.119)	0.881
	Maternal anxiety during pregnancy	-0.039 (-0.129 to 0.052)	0.408
	Maternal gestational weight gain (Kg/week)	-0.004 (-0.239 to 0.231)	0.971
	Air pollution exposure during pregnancy (µg/m^3)	-0.006 (-0.016 to 0.005)	0.299
Postnatal characteristics	Maternal smoking during first year of age	-0.055 (-0.157 to 0.048)	0.303
	Day care attendance during first year of age	0.075 (-0.072 to 0.223)	0.322
	Family pet ownership during first year of age	-0.031 (-0.098 to 0.037)	0.379
	Maternal anxiety during first year of age	0.047 (-0.033 to 0.126)	0.253
	Air pollution during first year of age (µg/m^3)	-0.009 (-0.021 to 0.004)	0.169
	Breastfeeding during first 6 months	-0.006 (-0.108 to 0.096)	0.907
	Early second-hand smoke exposure	-0.023 (-0.104 to 0.059)	0.588
Early-Childhood characteristics	Second-hand smoke exposure during age 1-8 y	-0.025 (-0.089 to 0.040)	0.458
	Air pollution during 1-7 years of age (µg/m^3)	-0.009 (-0.019 to 0.001)	0.074
	Lean mass at age 9 years (kg) †	0.124 (0.105 to 0.144)	0.000
	Fat mass at age 9 years (kg/2) †	-0.009 (-0.025 to 0.007)	0.277
	Current asthma at 7.5 years	-0.064 (-0.162 to 0.033)	0.203
	Skin Prick Test at 7.5 years	-0.001 (-0.103 to 0.100)	0.977
Adoles.	Smoking status at 14 years	0.094 (0.008 to 0.181)	0.038
	Age at peak height velocity in puberty (years)	0.003 (-0.021 to 0.027)	0.800
	Peak height velocity in puberty (cm/year)	0.000 (-0.022 to 0.022)	0.998

Abbreviations: FEV_1 = forced expiratory volume in one second; Adoles. = adolescence; Kg = kilogram; m = metre; µg = microgram; cm = centimetre.

*Educated to the General Certificate of Education level (school-leaving certificate) or lower, see **Error! Reference source not found.**.

Table S12. Crude associations of early-life characteristics with SD scores of FEV_1/FVC (scores adjusted for sex, age and height) at age 24 years (N=7545).

Stage	Factor	Difference in SD scores of FEV_1/FVC (95% CI)	P-value
Demographic, maternal and child characteristics	Overcrowding	-0.068 (-0.164 to 0.029)	0.175
	Gas cooking	-0.001 (-0.078 to 0.076)	0.978
	Rented housing	-0.140 (-0.248 to -0.031)	0.015
	Single Mother	-0.052 (-0.153 to 0.049)	0.323
	Low maternal education*	-0.095 (-0.156 to -0.034)	0.003
	Maternal history of asthma or allergy	-0.006 (-0.075 to 0.064)	0.873
	Family financial difficulties	-0.226 (-0.408 to -0.044)	0.018
	Parity (>= 1 siblings)	0.051 (-0.019 to 0.121)	0.157
Perinatal characteristics	Maternal perinatal body mass index (Kg/m^2)	-0.020 (-0.030 to -0.010)	0.000
	Maternal age at delivery > 28 years (the median)	0.068 (-0.004 to 0.140)	0.069
	Birthweight (Kg)	0.037 (-0.031 to 0.106)	0.291
	Pre-term delivery	-0.250 (-0.404 to -0.096)	0.002
	Caesarean section	-0.104 (-0.222 to 0.014)	0.089
	Maternal smoking during pregnancy	-0.198 (-0.296 to -0.100)	0.000
	Maternal anxiety during pregnancy	-0.042 (-0.137 to 0.053)	0.397
	Maternal gestational weight gain (Kg/week)	-0.067 (-0.305 to 0.172)	0.587
	Air pollution exposure during pregnancy ($\mu g/m^3$)	0.005 (-0.006 to 0.016)	0.404
Postnatal characteristics	Maternal smoking during first year of age	-0.189 (-0.298 to -0.081)	0.002
	Day care attendance during first year of age	0.009 (-0.130 to 0.149)	0.897
	Family pet ownership during first year of age	-0.013 (-0.079 to 0.054)	0.703
	Maternal anxiety during first year of age	-0.005 (-0.077 to 0.066)	0.880
	Air pollution during first year of age ($\mu g/m^3$)	0.000 (-0.015 to 0.015)	0.973
	Breastfeeding during first 6 months	-0.007 (-0.127 to 0.114)	0.911
	Early second-hand smoke exposure	-0.106 (-0.183 to -0.029)	0.010
Early-Childhood characteristics	Second-hand smoke exposure during age 1-8 y	-0.118 (-0.182 to -0.054)	0.001
	Air pollution during 1-7 years of age ($\mu g/m^3$)	0.002 (-0.007 to 0.012)	0.640
	Lean mass at age 9 years (kg) †	-0.063 (-0.084 to -0.042)	0.000
	Fat mass at age 9 years (kg/2) †	-0.027 (-0.046 to -0.008)	0.009
	Current asthma at 7.5 years	-0.238 (-0.349 to -0.127)	0.000
	Skin Prick Test at 7.5 years	-0.003 (-0.097 to 0.091)	0.952
Adoles.	Smoking status at 14 years	-0.101 (-0.188 to -0.014)	0.027
	Age at peak height velocity in puberty (years)	0.024 (0.002 to 0.046)	0.031
	Peak height velocity in puberty (cm/year)	-0.016 (-0.035 to 0.004)	0.115

Abbreviations: FEV1 = forced expiratory volume in one second; FVC = forced vital capacity; Adoles. = adolescence; Kg = kilogram; m = metre; µg = microgram; cm = centimetre.

*Educated to the General Certificate of Education level (school-leaving certificate) or lower, see **Error! Reference source not found.**.

Table S13. Crude associations of early-life characteristics with SD scores of FEF_{25-75} (scores adjusted for sex, age and height) at age 24 years (N=7545).

Stage	Factor	Difference in SD scores of FEF_{25-75} (95% CI)	P-value
Demographic, maternal and child characteristics	Overcrowding	-0.045 (-0.143 to 0.054)	0.378
	Gas cooking	-0.010 (-0.087 to 0.067)	0.802
	Rented housing	-0.102 (-0.201 to -0.003)	0.049
	Single Mother	-0.052 (-0.146 to 0.043)	0.290
	Low maternal education*	-0.084 (-0.143 to -0.026)	0.006
	Maternal history of asthma or allergy	0.007 (-0.061 to 0.076)	0.834
	Family financial difficulties	-0.252 (-0.424 to -0.079)	0.005
	Parity (>= 1 siblings)	0.090 (0.020 to 0.160)	0.014
Perinatal characteristics	Maternal perinatal body mass index (Kg/m^2)	0.001 (-0.009 to 0.011)	0.843
	Maternal age at delivery > 28 years (the median)	0.097 (0.031 to 0.163)	0.005
	Birthweight (Kg)	0.112 (0.056 to 0.168)	0.000
	Pre-term delivery	-0.302 (-0.470 to -0.135)	0.001
	Caesarean section	-0.102 (-0.215 to 0.012)	0.085
	Maternal smoking during pregnancy	-0.123 (-0.237 to -0.008)	0.044
	Maternal anxiety during pregnancy	-0.010 (-0.101 to 0.081)	0.825
	Maternal gestational weight gain (Kg/week)	-0.086 (-0.332 to 0.161)	0.499
	Air pollution exposure during pregnancy ($\mu g/m^3$)	-0.001 (-0.012 to 0.010)	0.858
Postnatal characteristics	Maternal smoking during first year of age	-0.160 (-0.271 to -0.048)	0.008
	Day care attendance during first year of age	0.068 (-0.060 to 0.195)	0.301
	Family pet ownership during first year of age	0.001 (-0.067 to 0.068)	0.982
	Maternal anxiety during first year of age	0.006 (-0.065 to 0.078)	0.866
	Air pollution during first year of age ($\mu g/m^3$)	-0.005 (-0.018 to 0.008)	0.434
	Breastfeeding during first 6 months	-0.035 (-0.152 to 0.081)	0.553
	Early second-hand smoke exposure	-0.069 (-0.151 to 0.013)	0.108
Early-Childhood characteristics	Second-hand smoke exposure during age 1-8 y	-0.103 (-0.171 to -0.035)	0.004
	Air pollution during 1-7 years of age ($\mu g/m^3$)	-0.003 (-0.013 to 0.006)	0.472
	Lean mass at age 9 years (kg) †	0.031 (0.010 to 0.052)	0.007
	Fat mass at age 9 years (kg/2) †	-0.002 (-0.020 to 0.016)	0.798
	Current asthma at 7.5 years	-0.229 (-0.317 to -0.142)	0.000
	Skin Prick Test at 7.5 years	0.003 (-0.099 to 0.105)	0.955
Adoles.	Smoking status at 14 years	-0.048 (-0.131 to 0.035)	0.262
	Age at peak height velocity in puberty (years)	0.007 (-0.015 to 0.029)	0.545
	Peak height velocity in puberty (cm/year)	-0.011 (-0.031 to 0.009)	0.293

Abbreviations: FEF_{25-75} = forced expiratory flow, midexpiratory phase; Adoles. = adolescence; Kg = kilogram; m = metre; µg = microgram; cm = centimetre.

*Educated to the General Certificate of Education level (school-leaving certificate) or lower, see **Error! Reference source not found.**.

FIGURES

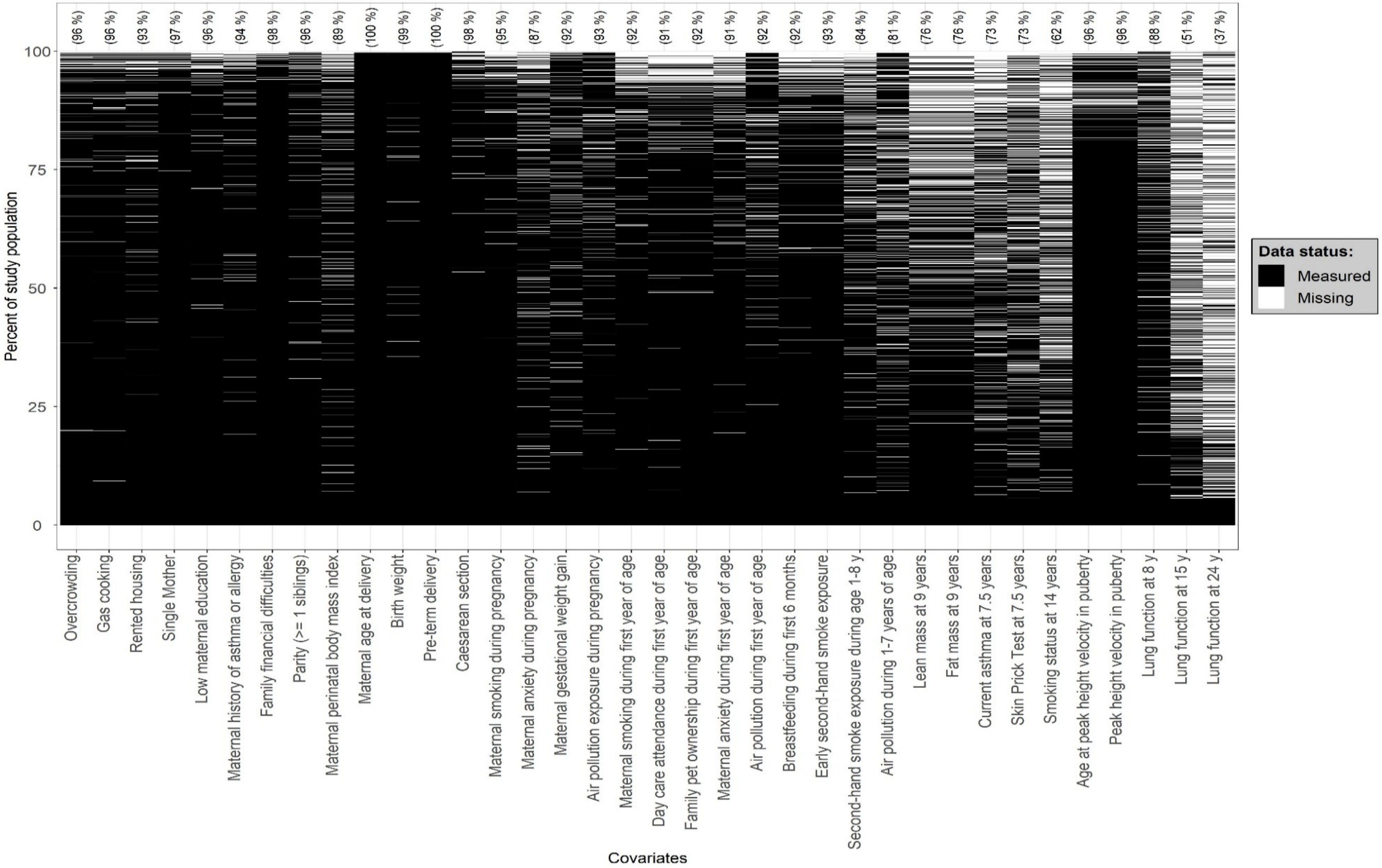

Figure S1. Layout of missing data among study population (N=7,545), with percent of observed data shown above corresponding characteristic's column

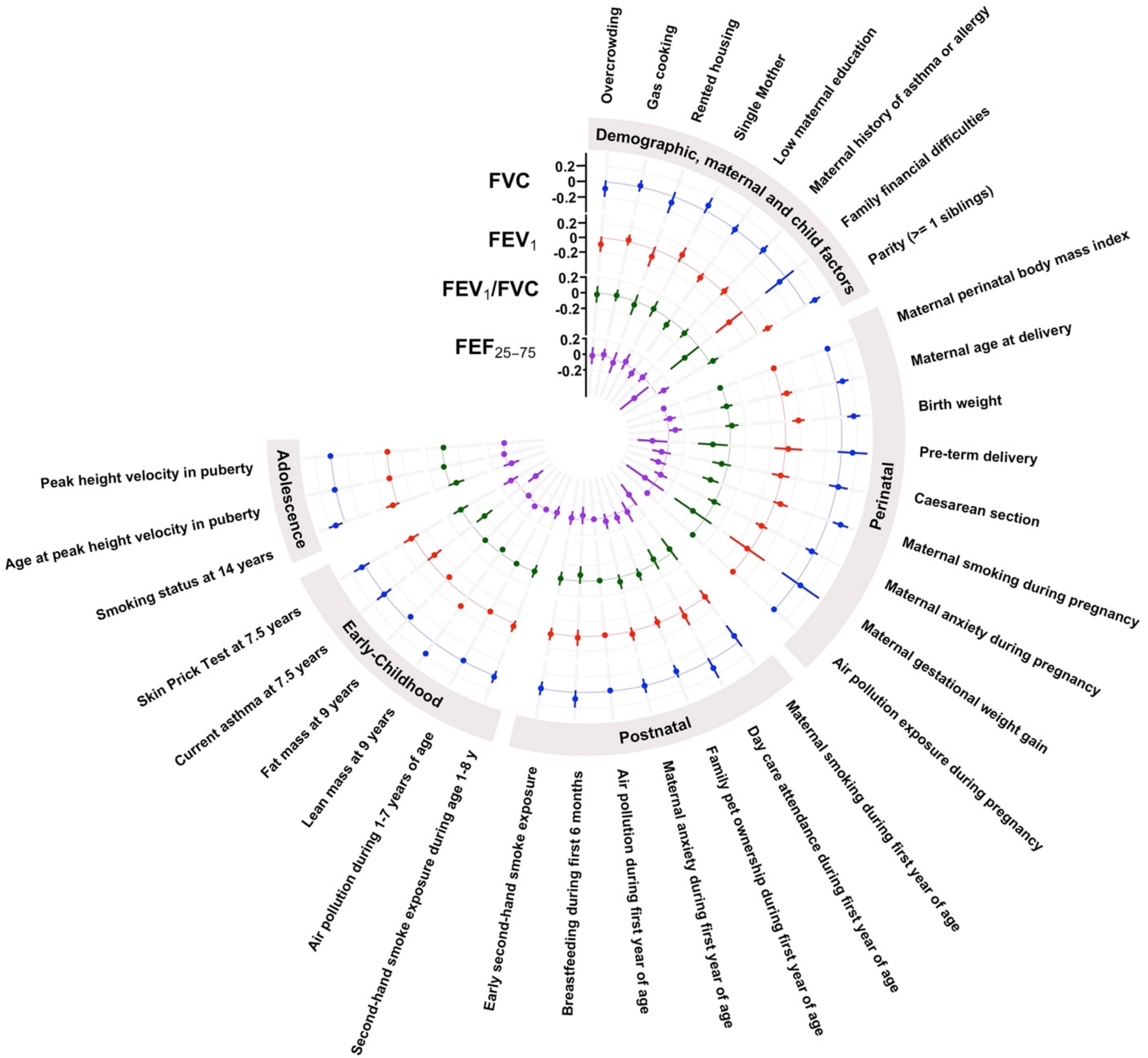

Figure S2. Circular plot of characteristics' association (point estimates and 95% confidence intervals) with measured (non-imputed) lung function parameters at age 24 years (N=2800). The raw data used for generating this plot are reported in Tables S6-S9.

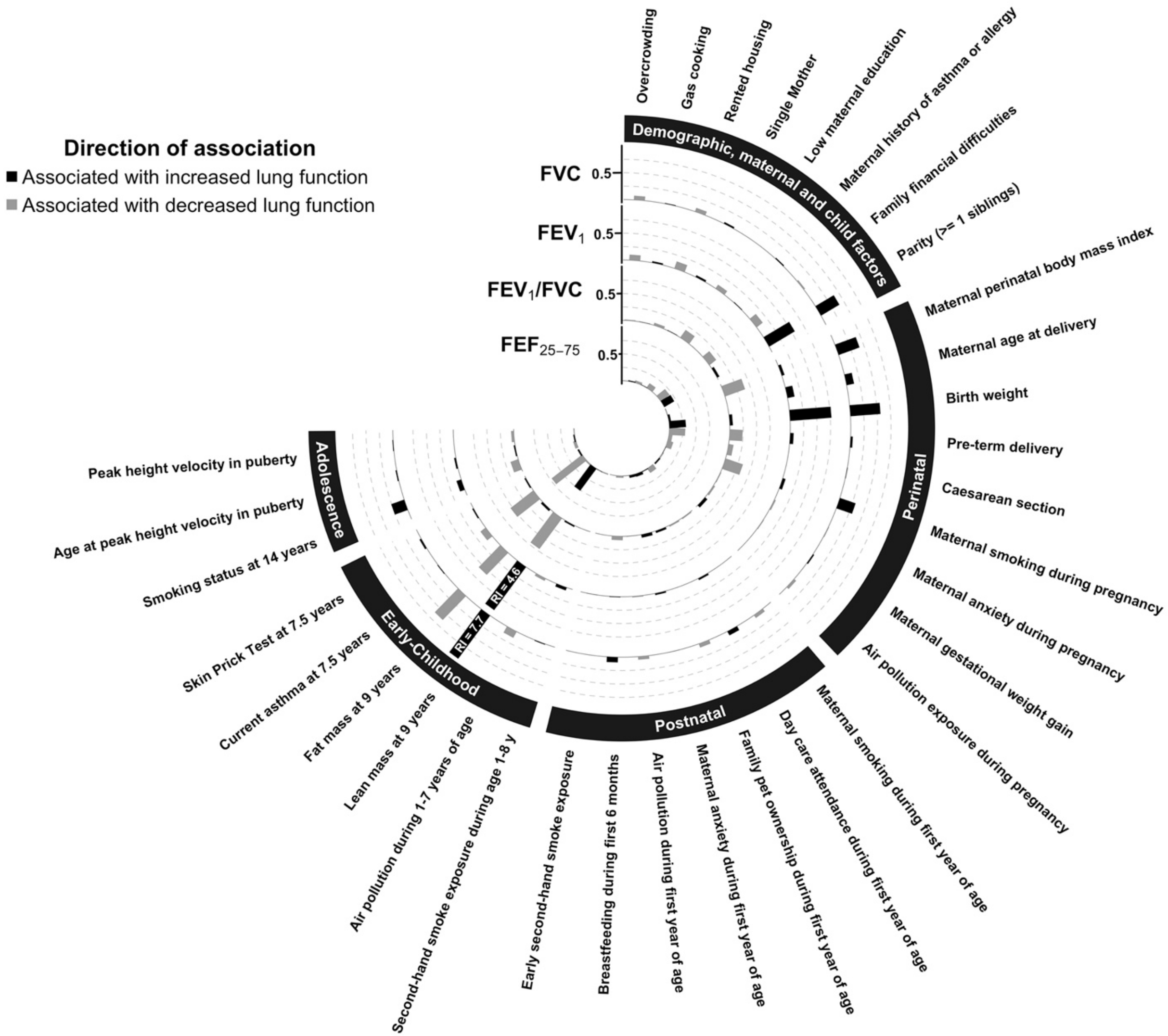

Figure S3 Circular plot of characteristics' relative importance (RI), on measured (non-imputed) lung function parameters at age 24 years (N=2800). Associations with higher and lower lung function were highlighted in black and grey colours respectively. Bars' height represents levels of RI, expressed in %, except for characteristics whose RI $> 1\%$, where exact RI values are displayed on their corresponding bars
