# Supplementary material for: Early-life and health behaviour influences on lung function in early adulthood
Source: Eur Respir J. 2023 Mar 2;61(3):2001316. doi: 10.1183/13993003.01316-2020 (PMC9978163; doi:10.1183/13993003.01316-2020)
Supplement: Supplementary file 2 [file ERJ-01316-2020.Shareable.pdf]

SHAREABLE PDF

Early-life and health behaviour influences on lung function in early adulthood

Osama Mahmoud [1,2,3], Raquel Granell[2,4], Gabriela P. Peralta [5], Judith Garcia-Aymerich [6,7,8], Deborah Jarvis [9,10], John Henderson[2,4,†] and Jonathan Sterne[2,4]

[1]Dept of Mathematical Sciences, University of Essex, Colchester, UK. [2]Population Health Sciences, Bristol Medical School, University of Bristol, Bristol, UK. [3]Dept of Applied Statistics, Helwan University, Cairo, Egypt. [4]MRC Integrative Epidemiology Unit (IEU), Bristol Medical School, University of Bristol, Bristol, UK. [5]Epidemiology, Biostatistics and Prevention Institute (EBPI), University of Zurich, Zurich, Switzerland. [6]ISGlobal, Barcelona, Spain. [7]Universitat Pompeu Fabra (UPF), Barcelona, Spain. [8]CIBER Epidemiología y Salud Pública (CIBERESP), Barcelona, Spain. [9]National Heart and Lung Institute, Imperial College, London, UK. [10]MRC-PHE Centre for Environment and Health, Imperial College, London, UK. [†]Deceased.

Corresponding author: Osama Mahmoud (o.mahmoud@essex.ac.uk)

Check for updates

Shareable abstract (@ERSpublications)
Perinatal characteristics (*e.g.* birth weight) and childhood characteristics (*e.g.* lean mass, fat mass and asthma at primary school age) had the most influence on lung function in early adulthood
https://bit.ly/3TggF1Q

Cite this article as: Mahmoud O, Granell R, Peralta GP, *et al*. Early-life and health behaviour influences on lung function in early adulthood. *Eur Respir J* 2023; 61: 2001316 [DOI: 10.1183/13993003.01316-2020].

This single-page version can be shared freely online.

Copyright ©The authors 2023.

This version is distributed under the terms of the Creative Commons Attribution Licence 4.0.

Received: 21 April 2020
Accepted: 11 Oct 2022

Abstract

Rationale Early-life exposures may influence lung function at different stages of the life course. However, the relative importance of characteristics at different stages of infancy and childhood are unclear.

Objectives To examine the associations and relative importance of early-life events on lung function at age 24 years.

Methods We followed 7545 children from the Avon Longitudinal Study of Parents and Children from birth to 24 years. Using previous knowledge, we classified an extensive list of putative risk factors for low lung function, covering sociodemographic, environmental, lifestyle and physiological characteristics, according to timing of exposure: 1) demographic, maternal and child; 2) perinatal; 3) postnatal; 4) early childhood; and 5) adolescence characteristics. Lung function measurements (forced vital capacity (FVC), forced expiratory volume in 1 s (FEV_1), FEV_1/FVC and forced expiratory flow at 25–75% of FVC) were standardised for sex, age and height. The proportion of the remaining variance explained by each characteristic was calculated. The association and relative importance (RI) of each characteristic for each lung function measure was estimated using linear regression, adjusted for other characteristics in the same and previous categories.

Results Lower maternal perinatal body mass index (BMI), lower birthweight, lower lean mass and higher fat mass in childhood had the largest RI (0.5–7.7%) for decreased FVC. Having no siblings, lower birthweight, lower lean mass and higher fat mass were associated with decreased FEV_1 (RI 0.5–4.6%). Higher lean mass and childhood asthma were associated with decreased FEV_1/FVC (RI 0.6–0.8%).

Conclusions Maternal perinatal BMI, birthweight, childhood lean and fat mass and early-onset asthma are the factors in infancy and childhood that have the greatest influence on early-adult lung function.
